# Bacterial dynamics of the plastisphere microbiome exposed to sub-lethal antibiotic pollution

**DOI:** 10.1186/s40168-024-01803-2

**Published:** 2024-05-24

**Authors:** Brune Joannard, Concepcion Sanchez-Cid

**Affiliations:** Université de Lyon, Université Claude Bernard Lyon 1, UMR CNRS 5557, UMR INRAe 1418, VetAgro Sup, Ecologie Microbienne, 69622 Villeurbanne, France

**Keywords:** Plastisphere, Sub-lethal, Antibiotics, Antibiotic resistance, Metagenomics, Surface water, Microplastics

## Abstract

**Background:**

Antibiotics and microplastics are two major aquatic pollutants that have been associated to antibiotic resistance selection in the environment and are considered a risk to human health. However, little is known about the interaction of these pollutants at environmental concentrations and the response of the microbial communities in the plastisphere to sub-lethal antibiotic pollution. Here, we describe the bacterial dynamics underlying this response in surface water bacteria at the community, resistome and mobilome level using a combination of methods (next-generation sequencing and qPCR), sequencing targets (16S rRNA gene, pre-clinical and clinical class 1 integron cassettes and metagenomes), technologies (short and long read sequencing), and assembly approaches (non-assembled reads, genome assembly, bacteriophage and plasmid assembly).

**Results:**

Our results show a shift in the microbial community response to antibiotics in the plastisphere microbiome compared to surface water communities and describe the bacterial subpopulations that respond differently to antibiotic and microplastic pollution. The plastisphere showed an increased tolerance to antibiotics and selected different antibiotic resistance bacteria (ARB) and antibiotic resistance genes (ARGs). Several metagenome assembled genomes (MAGs) derived from the antibiotic-exposed plastisphere contained ARGs, virulence factors, and genes involved in plasmid conjugation. These include *Comamonas, Chryseobacterium,* the opportunistic pathogen *Stenotrophomonas maltophilia*, and other MAGs belonging to genera that have been associated to human infections, such as *Achromobacter.* The abundance of the integron-associated ciprofloxacin resistance gene *aac(6’)-Ib-cr* increased under ciprofloxacin exposure in both freshwater microbial communities and in the plastisphere. Regarding the antibiotic mobilome, although no significant changes in ARG load in class 1 integrons and plasmids were observed in polluted samples, we identified three ARG-containing viral contigs that were integrated into MAGs as prophages.

**Conclusions:**

This study illustrates how the selective nature of the plastisphere influences bacterial response to antibiotics at sub-lethal selective pressure. The microbial changes identified here help define the selective role of the plastisphere and its impact on the maintenance of environmental antibiotic resistance in combination with other anthropogenic pollutants. This research highlights the need to evaluate the impact of aquatic pollutants in environmental microbial communities using complex scenarios with combined stresses.

Video Abstract

**Supplementary Information:**

The online version contains supplementary material available at 10.1186/s40168-024-01803-2.

## Background

Human activities have contributed to the dramatic global increase in antibiotic resistance in clinical and environmental settings [1, 2] despite the natural background levels of antibiotic resistance in the environment [3]. Urgent solutions are needed in order to preserve the efficacy of antibiotic therapy [3]. Anthropogenic activities lead to environmental pollution that might stimulate the development of antibiotic resistance in environmental reservoirs and the dissemination of this resistance to the human microbiome [4–7]. Antibiotics and their role as environmental pollutants have been widely studied, since they may impose a direct selective pressure for antibiotic resistance in environmental bacteria [8–11]. The anthropogenic uses of antibiotics and their incomplete elimination during wastewater treatment lead to a release of residual concentrations of these drugs into the environment [12, 13]. Antibiotics are often found in the environment at sub-inhibitory concentrations (i.e., too low to significantly inhibit overall community growth) [14]. However, environmental, sub-inhibitory levels of antibiotics can affect bacterial dynamics, select for antibiotic resistant bacteria (ARB) and antibiotic resistance genes (ARGs), and change the evolution of mobile genetic elements (MGEs) in the environment [10, 15, 16]. The presence of antibiotic residues in the environment may, therefore, increase the risk of antibiotic resistance dissemination in environmental settings that might consequently disseminate to the human microbiome.

In addition, non-antibiotic pollutants such as metals, biocides, non-antibiotic drugs, and microplastics are often found in the environment and may co-select for antibiotic resistance [17–20]. Plastics produced by humans are one of the primary sources of anthropogenic environmental pollution with 368 million tons produced in 2019 [21]. This microplastic pollution is smaller than 5 mm in size [22] and derived from the degradation of larger plastics or directly discharged into the environment by wastewater treatment plants, industries, and agricultural activities [23]. Microplastics are found ubiquitously in aquatic environments, including sea water and freshwater [24–26]. They provide a stable ecosystem for the microorganisms that are able to form biofilms on their surface. Bacterial community structure in this ecosystem, known as the “plastisphere” [27], varies substantially from that of the surrounding environment [28, 29]. Several studies have shown an enrichment of both ARB and ARGs in the plastisphere [30, 31]. Some explanations of this phenomenon are the colonization of the plastic surface by bacteria that carry antibiotic resistance genes and that proliferate in the biofilm (vertical transfer) [32] and an increased horizontal gene transfer of ARGs given the closer contact between bacteria in the biofilm and an increased ROS generation and cell permeability [32–35]. Thus, microplastics are a reservoir of antibiotic resistance that can transport and disseminate antibiotic resistance in the environment [31], where they can persist for long periods of time [27]. In addition, microplastics may be ingested by living organisms [36, 37] that could act as dissemination routes through the food chain [38]. Given the numerous routes microplastics and their associated plastisphere can disseminate through the environment and eventually to the human microbiome, they are considered to be a threat to human health [39].

Microplastics and antibiotics often coexist in environments such as urban water. Microplastics may adsorb a wide range of chemicals such as antibiotics, heavy metals, and other xenobiotics [39]. The adsorption of antibiotics onto plastic surfaces could increase their concentrations related to the surrounding environment and the selective pressure on the bacterial communities [40]. In other words, the presence of microplastics and their associated plastisphere could increase the magnitude of the antibiotic resistance selection that antibiotics induce in environmental communities [41–43]. On the other hand, bacteria in the plastisphere could be more tolerant to antibiotics than planktonic bacteria in surface waters [44]. Also, microplastic pollution could increase the persistence of antibiotics in the environment and their associated risks for human health [45]. Therefore, there are increasing concerns that the plastisphere could be involved in environmental antibiotic resistance, so that research is needed to understand the dynamics of the bacterial communities exposed to antibiotics in the plastisphere [39, 46].

The primary goal of this study was to explore the microbial ecology of the antibiotic-exposed plastisphere to determine whether the impact of microplastics on bacterial communities imposes an additional selective pressure that changes the outcome of antibiotic-induced selection. In addition, we aimed to understand the mechanisms involved in the response to microplastics and antibiotics individually and in combination at the community level (community dynamics and selection on putative ARB, i.e., bacterial genomes encoding genes related to antibiotic resistance) and the genetic level (selection and genetic context of ARGs). We hypothesized that communities in the plastisphere would be more tolerant to antibiotics and exhibit a reduced response to antibiotic pollution than freshwater bacterial communities not exposed to microplastics. Using a combination of sequencing targets (16S rRNA gene, short and long-read metagenomic and class 1 integron cassette sequencing), plasmid, bacteriophage and genome assembly-based approaches, gene screening in non-assembled reads, and gene quantification by qPCR, we evaluated the response to antibiotics at sub-lethal concentrations in a river water bacterial community in the presence and absence of microplastics.

## Materials and methods

### Experimental setup

One liter of urban river water from the Rhône river in Lyon was sampled on January 4, 2023 (45°45′08.3″N 4°50′11.3″E), in a polypropylene container and processed 30 min after collection. In order to reduce the amount of antibiotic residues and other xenobiotics potentially present in the samples, 500 ml of river water were autoclaved. One milliliter of river water containing bacteria was incubated in parallel in 9 ml of 1:10 TSB overnight at 25 °C and 185 rpm. Then, 0.5 ml of overnight culture was inoculated into 4.5 ml of sterile Rhône river water. Three conditions were prepared in triplicate: bacteria exposed to microplastics, bacteria exposed to antibiotics, and bacteria exposed to both microplastics and antibiotics. In addition, triplicate controls that were not polluted in the laboratory were included in the study to compare their evolution over time to that of polluted samples. Polystyrene was selected as a model of microplastics given its low biodegradability and frequent detection in environmental settings [21]. Approximately 100 spherical polystyrene particles with a diameter of 430 µm (Sigma-Aldrich) were added per sample. Ciprofloxacin and gentamicin were selected as antibiotic models given their incomplete removal during wastewater treatment [47] and their reported effects on antibiotic resistance selection at sub-inhibitory concentrations [15, 16, 48]. Gentamicin (Duchefa Biochemie) and ciprofloxacin (Sigma-Aldrich) were added together at 100 ng/ml each. The choice of this antibiotic concentration was based on a sub-inhibitory effect of both antibiotics at 100 ng/ml on river water bacteria in vitro (Figure S1 in Supplementary Information). Samples were incubated at 25 °C for 3 days and shaken to emulate river currents (185 rpm) before extracting DNA from bacteria in the plastisphere and in river water. In order to reduce the influence of external physiochemical factors, temperature was kept constant through the incubation and samples were protected from UV exposure.

### DNA extraction and estimation of bacterial community abundance by qPCR

Samples without microplastics were centrifuged at 2500 rpm for 10 min and the pellet was resuspended in 500 µl of the extraction buffer from the NucleoSpin Tissue Kit (Macherey–Nagel). Polystyrene beads were left to decant and, after completely removing all the surrounding water, they were resuspended in 500 µl of extraction buffer. Samples were transferred to a Lysis Matrix D tube (MP Biomedicals), heated for 5 min at 95 °C, and underwent beat-beating at 5.5 m/s for 30 s (twice for river water bacterial pellets and three times for bacteria in the plastisphere). Total genomic DNA was then extracted using the NucleoSpin Tissue Kit (Macherey–Nagel) according to manufacturer’s instructions.

In order to determine whether the added antibiotics had an inhibitory effect on environmental bacteria at the community level (i.e., significant inhibition of overall bacterial growth), the size of the bacterial communities in river water and the plastisphere with and without antibiotic exposure was estimated by quantifying the 16S rRNA gene by qPCR using the 341F (5′-CCTACGGGAGGCAGCAG- 3′) and 534R (5′-ATTACCGCGGCTGCTGGCA-3′) primers [49]. qPCR amplification was carried out using the CFX Duet Real-Time PCR System (Bio-Rad) in a 20 µl reaction volume containing QuantiNova SYBR Green PCR Master Mix (Qiagen), 0.75 µM of each primer, and 2 µl of DNA. Two non-template controls were included in the assay. Standard curves for all the assays were obtained using tenfold serial dilutions of a linearized plasmid pGEM-T Easy Vector (10^8^–10^3^ copies) containing the 16S rRNA gene of *Pseudomonas aeruginosa* PAO1. Cycling conditions for qPCR amplification were 95 °C for 2 min followed by 35 cycles of 95 °C for 5 s and 60 °C for 30 s. Melting curves were generating by increasing temperature from 60 °C to 95 °C after amplification. The reaction had an efficiency of 100% and a linearity R^2^ coefficient of 0.98.

### 16S rRNA gene sequencing and bioinformatic analysis of bacterial community composition

The V4 hypervariable region of the 16S rRNA gene was amplified using forward 515F (5’-TCGTCGGCAGCGTCAGATGTGTATAAGAGACAGGTGYCAGCMGCCGCGGTAA-3’) and reverse 806Rb (‘5-GTCTCGTGGGCTCGGAGATGTGTATAAGAGACAGGGACTACNVGGGTWTCTAAT-3’) primers with Illumina overhangs [50]. This amplicon (~ 250 bp) was chosen to optimize read overlap for the sequence merging step. DNA was amplified by PCR using the Platinum Taq DNA Polymerase (Invitrogen) and the following conditions: 94 °C for 2 min, 30 cycles of 94 °C for 30 s, 55 °C for 30 s and 72 °C for 30 s, and a final extension for 5 min at 72 °C. DNA libraries were prepared from amplified products using the Platinum Taq DNA Polymerase (Invitrogen) and the Nextera XT Index Kit V2 (Illumina) according to the Illumina’s protocol for amplicon sequencing library preparation. Paired-end sequencing (2 × 251 bp) of barcoded amplicons was performed using the MiSeq System and the MiSeq Reagent Kit v2 (Illumina) in the laboratory on our sequencer.

Sequences were treated using the DADA2 pipeline (version 1.16.0, R version 4.1.3) [51] to remove primers, trim the last 10 bases of the forward reads and the last 40 bases of the reverse reads based on read quality scores (Figures S2a and S2b in Supplementary Information), merge forward and reverse reads, remove chimeric reads, and obtain amplicon sequence variants (ASVs). The number of reads that went through each step of the pipeline are shown in Table S1 (Supplementary Information). ASVs were annotated taxonomically to the genus level using the Ribosomal Database Project (RDP trainset 18 downloaded from the DADA2 repository) [52]. Then, ASVs that had less than ten copies in total were removed. Sequencing depths obtained after sequence treatment and ASV removal are shown in Table S2 in Supplementary Material. The final ASV table with ASV abundance and taxonomic annotation, as well as a file with ASV sequences, have been uploaded as Additional Files 1 and 2, respectively. Statistical differences in ASV abundance between conditions were determined using the DESeq2 package in R (version 1.34.0) [53]. Log2FoldChange values were adjusted using the Approximate Posterior Estimation for generalized linear model in the apeglm package in R (version 1.16.0). Differences were defined based on a *p-*value < 0.05 and a log2FoldChange ≥ 2.

### Metagenomic sequencing and bioinformatic analyses

Metagenomics libraries were prepared from ≤ 1 ng of DNA using the Nextera XT Library Prep Kit and Indexes (Illumina) as detailed in Illumina’s “Nextera XT DNA Library Prep Kit” reference guide, using 12 amplification cycles for the indexing PCR. DNA sequencing was performed using the MiSeq System and the MiSeq Reagent Kit v2 (Illumina) in the laboratory on our sequencer. The same samples were re-sequenced in the laboratory on our sequencer using the MiniON, R10.4.1 flow cells, and the Native Barcoding Kit 24 V14 (Oxford Nanopore Technologies) to obtain longer reads. Six samples were barcoded per run, since DNA input was too low to run them separately. Long-read metagenomics libraries were prepared as detailed in Oxford Nanopore’s “Ligation Sequencing gDNA—Native Barcoding Kit 24 V14” protocol.

Short reads sequenced using the MiSeq System were quality-filtered according to the criteria described by Minoche et al. [54]. Sequencing depths of short and long reads are shown in Table S2 in Supplementary Material. Forward and reverse short reads were concatenated and blasted against the CARD amino acid database (version 3.2.8) [55] using Diamond [56], which translates nucleotide queries prior to blasting, in order to determine the abundance of total ARGs in the metagenomic reads. The obtained results were filtered at a minimum amino acid identity of 60%, a minimum length of 33 amino acids, and a maximum e-value of 10^−5^. The best hit was used, singletons were removed, and gene abundance was normalized by sequencing depth.

Quality-filtered short reads were co-assembled using MEGAHIT [57] and reads were mapped onto the contigs using Bowtie2 [58]. Profiles were created for each individual sample and merged using the anvi’o [59] metagenomic workflow (anvi’o version 7.1). The assembled contigs were blasted against the CARD antibiotic gene database to identify ARGs. Results were filtered at an amino acid identity percentage of 60%, 100 amino acid length, and an e-value of 10^−5^. The best hit was used. Finally, contigs were binned based on their differential coverage across samples using anvi’o, and the bins were refined based on differential coverage and sequence composition. Bins with < 50% completion and ≥ 10% redundancy were discarded, since they were considered low-quality metagenome assembled genome (MAG) drafts according to Bowers et al. [60]. Obtained bins had a completion from 78.9 to 100% and a redundancy from 2.8 to 7% (Table S3 in Supplementary Information). In parallel, two hybrid genome assembly methods were used on long and short reads. Quality-filtered short reads were co-assembled to long reads obtained from the Oxford Nanopore sequencing using Unicycler [61] or OPERA-MS without reference-guided binning [62] and MAGs were obtained using the anvi’o metagenomic workflow as described above. A single MAG of 100% completion and 0% redundancy was obtained from the Unicycler assembly, whereas 9 MAGs with a completion from 54.9 to 100% and a redundancy of 0 to 7% were obtained from the OPERA-MS hybrid assembly (Figure S3 in Supplementary Information).

In order to identify mechanisms related to a potential risk for human health, the MAGs were annotated using COG 2020, pfam v32 and SEED to identify type IV secretion system genes and virulence genes were identified in the MAGs using the BV-BRC database https://www.bv-brc.org/ [63]. Plastic degradation genes were identified using the online plastic degradation database PlasticDB https://plasticdb.org/ [64]. To determine the genetic context of ARGs, the PlasX machine-learning approach [65] was used to distinguish between chromosomal and plasmid contigs.

### Identification of antibiotic resistance genes in plasmids and bacteriophages

Plasmid reads were assembled from long and short reads using metaplasmidSPAdes (SPAdes version 3.15.5) [66] to identify changes in the plasmidome under antibiotic and/or microplastic pollution. Reads were co-assembled both altogether and per condition and ARGs were identified in plasmid contigs using CARD as described above (amino acid identity > 60% and alignment length > 150 amino acids).

Viral contigs were obtained from long and short reads using metaviralSPAdes (same SPAdes version) [67]. Reads from each sample were mapped onto the viral contigs using bowtie2 and ARGs were identified using CARD as described above (amino acid identity > 60% and alignment length > 150 amino acids). The taxonomy and bacterial hosts of ARG-containing viral contigs was determined using PhageScope [68]. In order to determine whether viral contigs were integrated in the MAGs, all obtained MAGs (regardless of the assembly approach) were blasted against the contigs (nucleotide blast). Blast results were filtered at > 99% nucleotide identity and > 1000 nucleotide alignment length.

### Evaluation of class 1 integron abundance and cassette array composition

Class 1 integron abundance was estimated by a qPCR amplification of the integrase *intI1* gene using HS463a (5′-CTGGATTTCGATCACGGCACG-3′) and HS464 (5′- ACATGCGTGTAAAT-CATCGTCG-3′) primers following the protocol and the amplification conditions described above (annealing temperature = 60 °C). Standards were obtained from freshwater DNA and cloned and transformed using the TOPO TA cloning Kit (Thermo Fisher Scientific). The reaction had an efficiency of 90.5% and a linearity *R*^2^ coefficient of 0.998.

Class 1 integron cassettes are often missed using metagenomic sequencing approaches. Therefore, in order to increase sensitivity and determine the composition of class 1 integron cassette arrays, they were amplified by PCR and sequenced. Both clinical and pre-clinical cassettes were evaluated in this study. Clinical class 1 integron cassettes were amplified using HS458 (5’-GTTTGATGTTATGGAGCAGCAACG-3’) and HS459 (5’-GCAAAAAGGCAGCAATTATGAGCC-3’) primers as described by Holmes et al. [69]. Pre-clinical class 1 integron cassettes were amplified using MRG284 (5’-GTTACGCCGTGGGTCGATG-3’) and MRG285 (5′-CCAGAGCAGCCGTAGAGC-3′) primers as described by Gillings et al. [70]. Then, amplicons were cleaned-up using AMPure XP beads (Beckman-Coulter) and sequenced using the MiSeq System in the laboratory on our sequencer as described for metagenomic reads. Reads were trimmed using the Fastq Quality Trimmer tool of the FASTX-Toolkit. Nucleotides that did not meet a minimum quality score of Q20 were trimmed from the sequences and sequences shorter than 100 nucleotides after trimming were removed. Sequencing depths obtained after sequence treatment are shown in Table S2 in Supplementary Material. Then, reads were concatenated and blasted against the CARD database as described for short read metagenomic reads to determine the overall ARG content of pre-clinical and clinical class 1 integron cassettes, identify ciprofloxacin and gentamicin resistance genes to target by qPCR, and determine whether the ARGs present in the MAGs are potentially associated to class 1 integrons.

### Gentamicin and ciprofloxacin resistance gene abundance

The abundance of a ciprofloxacin resistance gene (*aac(6’)-Ib-cr)* and a gentamicin resistance gene (*aac(6’)-IIc)* identified in class 1 integrons as well as that of the plasmid-borne ciprofloxacin resistance gene *qnrB* was determined in the freshwater and the plastisphere microbiomes by qPCR. Standards were obtained from freshwater DNA and cloned and transformed using the TOPO TA cloning Kit (Thermo Fisher Scientific). DNA was amplified as detailed above. The *aac(6’)-Ib-cr* gene was amplified using forward 5’-TTGCGATGCTCTATGAGTGGCTA-3’ and reverse 5’-CTCGAATGCCTGGCGTGTTT-3’ primers [71] and an annealing temperature of 57 °C. The reaction had an efficiency of 85.5% and a linearity *R*^2^ coefficient of 0.998. The *qnrB* gene was amplified using forward 5’-CTTCACACATTGCGATCTGAC’-3’ and reverse 5’- CAACGATGCCTGGTAGTTGT-3’ primers [72] and an annealing temperature of 60 °C. The reaction had an efficiency of 98.4% and a linearity *R*^2^ coefficient of 0.998. The *aac(6’)-IIc* gene was amplified using forward 5’-CGACCCGACTCCGAACAA-3’ and reverse 5’-GCACGAATCCTGCCTTCTCA-3’ primers [73] and an annealing temperature of 60 °C. The reaction had an efficiency of 93.6% and a linearity *R*^2^ coefficient of 1. The number of copies of each gene were normalized by the copies of the 16S rRNA gene per sample.

### Statistical analyses

All statistical analyses on metagenomic and qPCR data were carried out using GraphPad Prism 9. Normal distribution was tested using the Shapiro–Wilk test. Normally-distributed data were analyzed using one-way ANOVA and Tukey’s multiple comparison tests. Data that did not follow a normal distribution were analyzed using the Kruskal–Wallis and Dunn’s multiple comparison tests. A *p*-value lower than 0.05 was regarded as significant. Regarding multiple comparison tests, only significant differences are shown.

## Results

### Dynamics of bacterial community response and putative ARB selection

**Fig. 1:**
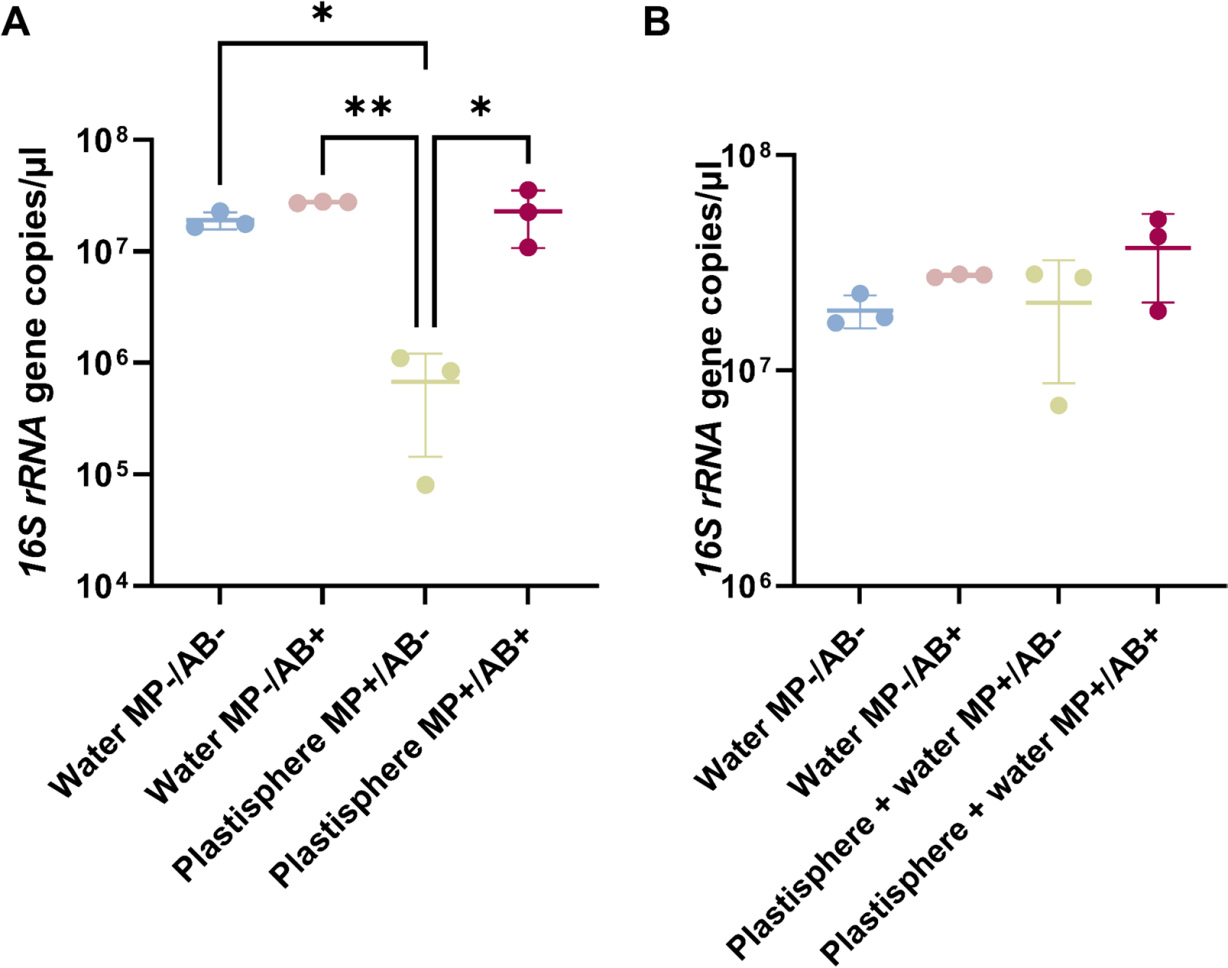
Dynamics in bacterial biomass in freshwater bacteria exposed to antibiotics, microplastics or both. **A** Comparison between freshwater bacteria non-exposed to microplastics and bacteria in the plastisphere (ANOVA *p-*value = 0.004). **B** Comparison between freshwater bacteria non-exposed to microplastic and total biomass (freshwater and plastisphere bacteria) in samples exposed to microplastics (ANOVA *p-*value = 0.2). Only significant pairwise comparisons (*p*-value < 0.05) are shown. Bacterial biomass was estimated by a qPCR of the 16S rRNA gene. MP-/AB- non-polluted freshwater controls, MP-/AB + antibiotic-exposed freshwater, MP + /AB- non-antibiotic-exposed plastisphere, MP + /AB + antibiotic-exposed plastisphere. *n* = 3

Bacterial biomass in the plastisphere non-exposed to antibiotics—estimated by a qPCR of the 16S rRNA gene—was lower (*p*-value < 0.05) than that of the antibiotic-exposed plastisphere and of freshwater bacterial communities non-exposed to microplastics, both in the presence and absence of antibiotics (Fig. 1A). On the other hand, the abundance of bacterial communities in the antibiotic-exposed plastisphere was similar to that of freshwater bacterial communities (Fig. 1A). In order to determine whether the total bacterial biomass in the samples was comparable, the biomass from samples in the plastisphere was added to that of the surrounding water of those samples. No significant differences in terms of total biomass were detected between samples exposed and not exposed to microplastics (Fig. 1B). Antibiotic levels were, therefore, considered as sub-lethal both in freshwater and in the plastisphere.

**Fig. 2:**
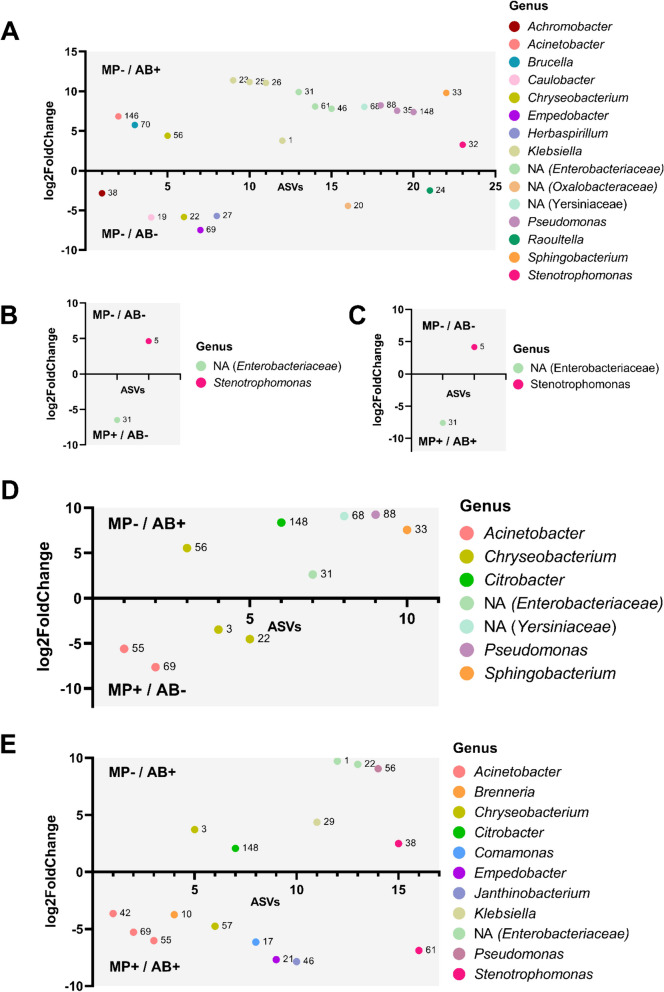
Significant differences (*p-*value < 0.05) in ASV abundance between experimental conditions. **A** Non-polluted water (MP-/AB-) versus antibiotic-polluted water (MP-/AB +). **B** Non-polluted water (MP-/AB-) versus plastisphere in the absence of antibiotics (MP + /AB-). **C** Non-polluted water (MP-/AB-) versus antibiotic-polluted plastisphere (MP + /AB +). **D** Antibiotic-polluted water (MP-/AB +) versus plastisphere in the absence of antibiotics (MP + /AB-). **E** Antibiotic-polluted water (MP-/AB +) versus antibiotic-polluted plastisphere (MP + /AB +). Statistical analyses on ASV abundances obtained from the sequencing of the 16S rRNA gene were performed using the DESeq2 package in R. Colors represent taxonomic affiliation of each ASV at the genus level. Labels represent the unique number associated to each ASV to help identify ASV variants from the same genus. No significant differences in ASV abundance were found between the antibiotic-exposed and the non-antibiotic-exposed plastisphere. *n* = 3

Overall bacterial community composition evaluated by the sequencing of the 16S rRNA gene was influenced by the exposure to antibiotics, microplastics, and both together (Figure S3 in Supplementary Information). A shift in community composition was observed between the antibiotic-exposed freshwater and plastisphere microbiomes. Antibiotic exposure at sub-lethal levels alone had the highest impact on freshwater bacterial community composition. A significant shift of bacterial community composition at the ASV level was found in freshwater bacteria exposed to antibiotics and had 16 ASVs that were higher (*p*-value < 0.05) and 7 ASVs that were significantly lower than in non-polluted controls (Fig. 2A). The ASVs that had a higher abundance in the antibiotic-exposed freshwater microbiome belonged to several genera including *Acinetobacter, Brucella, Chryseobacterium, Klebsiella, Pseudomonas, Sphingobacterium*, and *Stenotrophomonas.* The ASVs with a decreased abundance upon antibiotic exposure belonged to *Achromobacter, Caulobacter, Chryseobacterium, Empedobacter, Herbaspirillum,* and *Raoultella.* On the other hand, only one ASV from *Stenotrophomonas* had a lower relative abundance in the plastisphere than in non-polluted freshwater controls, whereas one ASV (not annotated to the genus level) belonging to the *Enterobacteriaceae* family was more abundant in the plastisphere. This was the case for both the antibiotic-exposed and non-antibiotic-exposed plastisphere (Fig. 2B, C). In addition, no significant differences were found between bacterial communities in the plastisphere exposed and not exposed to antibiotics.

Regarding the differences in community composition between antibiotic-exposed freshwater and non-antibiotic-exposed plastisphere, six ASVs belonging to several genera including *Chryseobacterium, Citrobacter, Pseudomonas,* and *Sphingobacterium* were found at a higher abundance in antibiotic-exposed freshwater communities than in the non-antibiotic-exposed plastisphere (Fig. 2D). On the other hand, four ASVs belonging to *Acinetobacter* and *Chryseobacterium* were more abundant in the plastisphere. Furthermore, a shift in the response to antibiotics was observed in the plastisphere with nine ASVs belonging to *Acinetobacter, Brenneria, Chryseobacterium, Comamonas, Empedobacter, Janthinobacterium,* and *Stenotrophomonas* showing a higher abundance in the antibiotic-exposed plastisphere and six ASVs related to *Chryseobacterium, Citrobacter, Klebsiella, Enterobacteriaceae, Pseudomonas,* and *Stenotrophomonas* showing a higher abundance in freshwater communities (Fig. 2E). Finally, two ASVs belonging to the *Acinetobacter* genus were consistently more abundant in the plastisphere than in antibiotic-exposed freshwater communities (Fig. 2D,E), whereas an ASV related to *Citrobacter* consistently showed a lower abundance in the plastisphere. However, different ASVs belonging to the same genera (*Acinetobacter, Chryseobacterium* and *Stenotrophomonas*) had different changes in abundance upon exposure to anthropogenic pollution depending on the nature of the pollutant (antibiotics, microplastics, or both) (Fig. 2A-E). These results indicate the presence of bacterial subpopulations that respond differently to these pollutants.

**Fig. 3:**
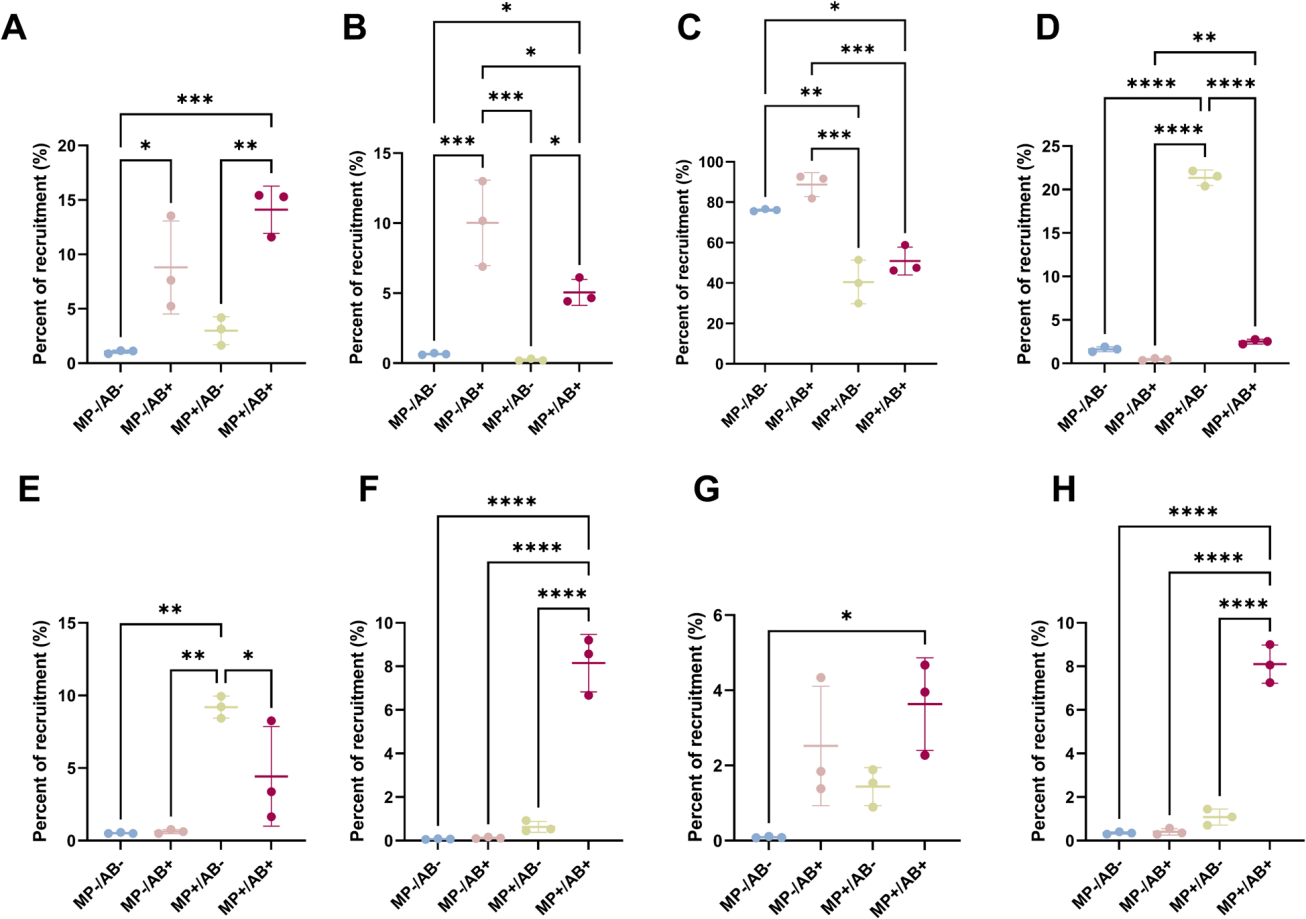
Relative abundance of the MAGs obtained from the short-read co-assembly and from the hybrid co-assembly of short and long reads. **A** *Achromobacter sp. 002902905* (MEGAHIT short-read assembly). **B** *Herbaspirillum* (OPERA-MS hybrid assembly). **C** *Stenotrophomonas maltophilia* (Unicycler hybrid assembly). **D** *Aeromonas* (OPERA-MS hybrid assembly). **E** *Acinetobater* (MEGAHIT short-read assembly). **F** *Comamonas* (OPERA-MS hybrid assembly). **G** *Chryseobacterium* (OPERA-MS hybrid assembly). **H** *Enterobacteriaceae* (OPERA-MS hybrid assembly). The percent of recruitment represents the percentage of reads from a sample that maps onto a MAG and is thus normalized by sequencing depth. ANOVA *p*-values: 0.0008 (**A**), 0.0002 (**B**), 0.0001 (**C**), 0001 (**D**), 0.0009 (**E**), < 0.0001 (**F**), 0.016 (**G**), < 0.0001 (**H**). Only pairwise comparisons with *p*-value < 0.05 are shown. *n* = 3

An assembly-based metagenomic approach was used to obtain a better understanding of the bacterial dynamics underlying the exposure to pollutants in the freshwater and the plastisphere communities. The hybrid assembly from long and short reads using the OPERA-MS assembler provided the higher number of MAGs (9) (Table S3 in Supplementary Information). The five MAGs obtained from the assembly of short reads were assigned to *Acinetobacter, Achromobacter sp. 002902905, Aeromonas, Herbaspirillum,* and *Stenotrophomonas maltophilia* and had a completion ranging from 78.9 to 100% and a redundancy of 2–7% (Table S3 in Supplementary Material). All these MAGs, except *Acinetobacter,* as well as five more associated to *Klebsiella, Comamonas, Chryseobacterium,* and two bacteria from the *Enterobacteriaceae* family were obtained using the OPERA-MS hybrid assembler. These MAGs had a completion from 54.9 to 100% and a redundancy of 0 to 7% (Table S3 in Supplementary Material). Finally, one single MAG associated to *Stenotrophomonas maltophilia* was found in the reads assembled using the Unicycler hybrid assembler. This MAG had a completion of 100%—similarly to the ones obtained using the other two approaches—and a redundancy of 0%, whereas the redundancy of the MAG obtained from short reads was of 4.2% and that of the MAG obtained using OPERA-MS was of 7% (Table S3 in Supplementary Material). Although OPERA-MS showed an overall better performance than the short-read assembly or the Unicycler hybrid assembler, it missed the *Acinetobacter* bin obtained using short reads and a complete, single-strain (0% redundancy) *Stenotrophomonas maltophilia* genome was obtained using Unicycler. In addition, when the same MAG was obtained using different assembly approaches, MAG abundance trends were similar regardless of the method used (Fig. 3 shows the abundance of the highest quality MAGs and Figures S4 and S5 show the abundance of the same MAGs obtained using other assembly approaches).

Different dynamics were observed during antibiotic and microplastic exposure (Fig. 3). All ARG-containing contigs identified in these MAGs were associated to chromosomes according to PlasX. The *Achromobacter* (Fig. 3A) and *Herbaspirillum* (Fig. 3B) MAGs were significantly more abundant in antibiotic-exposed samples. For *Achromobacter,* the increase in abundance was observed both in freshwater and in the plastisphere, whereas the abundance of *Herbaspirillum* increased more in the antibiotic-exposed freshwater communities. These MAGs contained chromosomally-encoded genes from efflux pumps operons involved in the resistance to ciprofloxacin and gentamicin (Table 1); some of these genes were found in class 1 integron cassette array sequences. Other ARGs mainly involved in antibiotic efflux (Table S4 in Supplementary Material) and genes related to virulence and conjugation (Table S5 in Supplementary Material) were also found in these MAGs. In addition, the *Stenotrophomonas maltophilia* MAG was predominant in all the samples (Fig. 3C), and it was the only MAG obtained from the hybrid assembly of short and long reads using Unicycler (Table S3 in Supplementary Information), with a completion of 100% and a redundancy of 0%. The abundance of the *Stenotrophomonas maltophilia* MAG was significantly reduced in the plastisphere, and it reached its highest upon antibiotic exposure in freshwater. In addition to the integron-associated *smeD* and *oqxAB* efflux mechanisms involved in resistance to ciprofloxacin (Table 1), this MAG contained a *qnrB71* ciprofloxacin resistance gene and a *aph(3’)-IIc* gentamicin resistance gene that was found in the sequences from the class 1 integron cassettes. Although *qnrB71* and *oqxAB* are known plasmid-borne ciprofloxacin resistance determinants, the contigs that contained these genes were predicted to be chromosomal and they were not found in plasmid contigs (Table S6 in Supplementary Information). Other genes related to antibiotic efflux and a beta-lactamase were also found in the MAG from *Stenotrophomonas maltophilia* in addition to genes related to conjugation and virulence.

**Table 1:** Characterization and genetic context of the genes related to ciprofloxacin and gentamicin resistance found in the MAGs

MAG	ARGs	CPX resistance	GM resistance	Full operon	Resistance mechanism	Found in integron reads
*Acinetobacter*	*abeM*	Yes	No	Yes	Antibiotic efflux	No
	*adeFGH*	Yes	No	Yes	Antibiotic efflux	No
*Achromobacter sp002902905*	*adeF*	Yes	No	No	Antibiotic efflux	Yes
	*axyXY-OprZ*	Yes	Yes	Yes	Antibiotic efflux	Yes
	*mexB*	Yes	No	No	Antibiotic efflux	No
*Aeromonas*	*acrB*	Yes	No	No	Antibiotic efflux	Yes
	*axyXY-OprZ*	Yes	Yes	No	Antibiotic efflux	Yes
*Herbaspirillum*	*adeF*	Yes	No	No	Antibiotic efflux	Yes
	*mexD*	Yes	Yes	No	Antibiotic efflux	Yes
	*axyY*	Yes	Yes	Yes	Antibiotic efflux	No
	*smeE*	Yes	No	No	Antibiotic efflux	Yes
	*acrD*	No	Yes	Yes	Antibiotic efflux	No
*Stenotrophomonas maltophilia*	*smeDEF*	Yes	No	Yes	Antibiotic efflux	Yes
	*aph(3')-IIc*	No	Yes	-	Antibiotic inactivation	Yes
	*qnrB71*	Yes	No	-	Antibiotic target protection	No
	*oqxAB*	Yes	No	Yes	Antibiotic efflux	Yes
*Comamonas*	*oqxAB*	Yes	No	Yes	Antibiotic efflux	Yes
	*acrB*	Yes	No	No	Antibiotic efflux	Yes
*Chryseobacterium*	-	-	-	-	-	-
*Enterobacteriaceae*	*acrD*	No	Yes	Yes	Antibiotic efflux	No
	*qnrB57*	Yes	No	-	Antibiotic target protection	No
	*acrAB*	Yes	No	Yes	Antibiotic efflux	Yes
	*cpxA*	No	Yes	Yes	Antibiotic efflux	Yes

Results obtained from blasting MAG sequences against the CARD antibiotic resistance gene database.

On the other hand, the *Aeromonas* (Fig. 3D) and *Acinetobacter* (Fig. 3E) MAGs were significantly more abundant in the plastisphere not exposed to antibiotics than in any other samples. These MAGs contained chromosomally encoded genes related to ciprofloxacin resistance but not to gentamicin resistance. The genes in *Acinetobacter* were not found in the sequences from the class 1 integron cassettes, whereas the *acrB* gene in the *Aeromonas* MAG was. The other ARGs found in this MAGs were mainly related to antibiotic inactivation (beta-lactamases were present in both MAGs and an aminoglycoside nucleotidyltransferase was detected in the *Acinetobacter* MAG). Both MAGs contained genes related to conjugation, although genes related to virulence were only detected in *Aeromonas.* Moreover, none of these two MAGs (or any other MAG analyzed in this study) contained polystyrene degradation genes, although a low-density polyethylene degradation gene was found in the *Acinetobacter* MAG. Three MAGs showed an increased relative abundance in the antibiotic-exposed plastisphere: *Comamonas* (Fig. 3F), *Chryseobacterium* (Fig. 3G), and a MAG of unknown genus belonging to the *Enterobacteriaceae* family (3H). This increase was significant compared to any other condition in the case of *Comamonas* and *Enterobacter*. Ciprofloxacin resistance genes found in class 1 integrons (*oqxAB* and *acrB*) were found in the *Comamonas* MAG, whereas the *Enterobacter* MAG contained resistance mechanisms against ciprofloxacin (*qnrB57, acrAB)* and gentamicin (*cpxA, acrD).* On the other hand, a higher variability is observed in the relative abundance of the *Chryseobacterium* MAG in polluted samples, and no antibiotic resistance genes were found in this MAG, suggesting its increased relative abundance under pollution could be due to other mechanisms. All these MAGs contained virulence and type IV secretion genes and lacked plastic degradation genes. Finally, two more MAGs associated to the *Enterobacteriaceae* family and *Klebsiella* were identified, and both of them showed a decreased abundance under both antibiotic and microplastic pollution (Figure S6 in Supplementary Information).

### Dynamics of ARG selection and mobilization potential

**Fig. 4:**
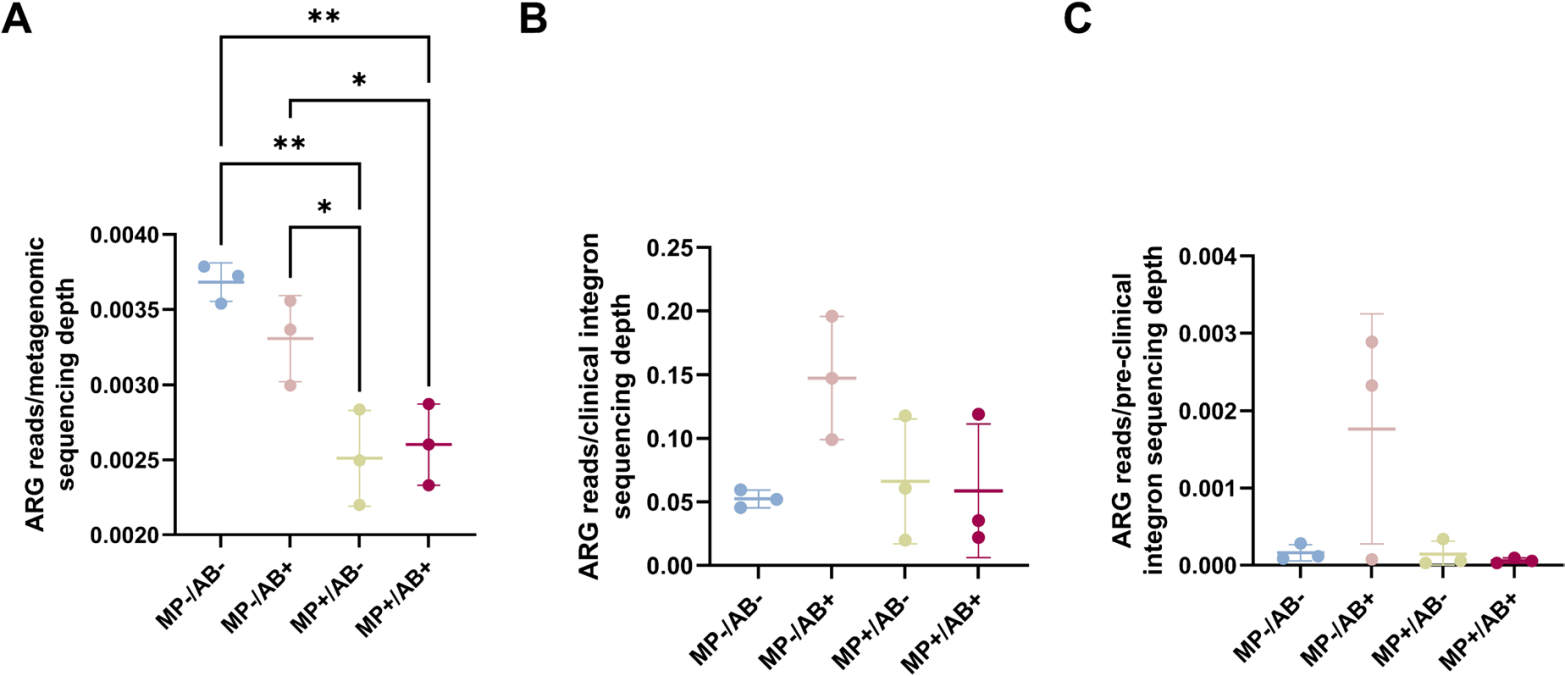
Changes in ARG load in the metagenomes (**A**) and in clinical (**B**) and pre-clinical (**C**) integron cassette arrays from freshwater bacteria exposed to antibiotics, microplastics, and both combined. ARG load was determined by blasting non-assembled metagenomic sequences, clinical and pre-clinical integron sequences against the CARD antibiotic resistance database. ARG abundance is normalized by sequencing depth. ANOVA *p*-values: 0.0015 (**A**), 0.16 (**B**), 0.08 (**C**). Only pairwise comparisons with *p*-value < 0.05 are shown. *n* = 3

**Fig. 5:**
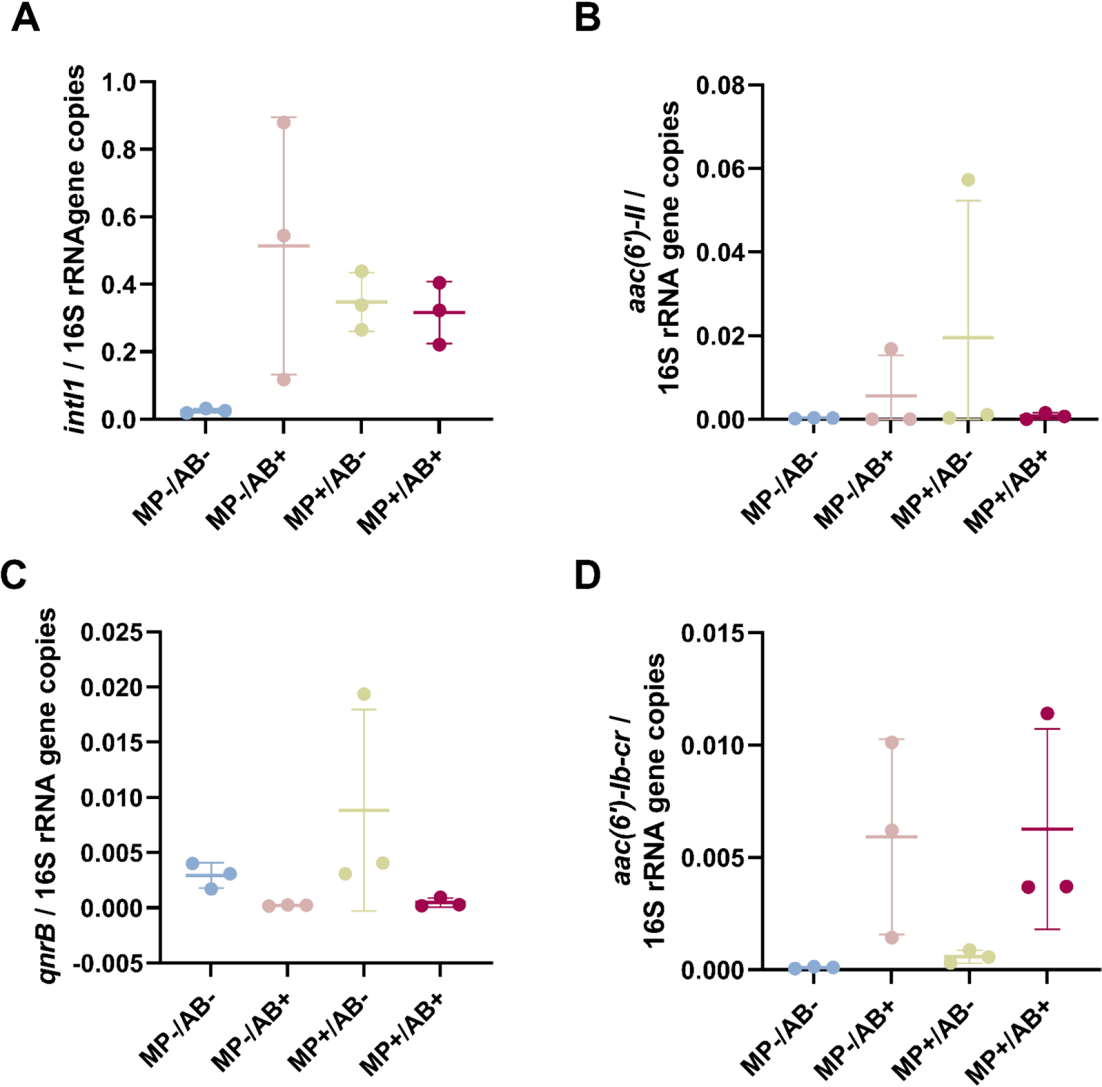
Relative abundance of class 1 integrons (**A**), the *aac(6()-II* gentamicin resistance gene (**B**), and the *qnrB* (**C**) and *aac(6’)-Ib-cr* (**D**) ciprofloxacin resistance genes in freshwater bacteria exposed to antibiotics, microplastics and both combined. Gene abundance was determined by qPCR and normalized by 16S rRNA gene abundance. ANOVA *p*-values: 0.09 (**A**), 0.15 (**C**). Kruskal–Wallis *p*-values: 0.07 (**B**), and 0.002 (**D**). *n* = 3

A decrease of total ARG load in the non-assembled metagenomic reads was observed in the plastisphere compared to freshwater, both in the presence and absence of antibiotics (Fig. 4A). A slight increase in the proportion of ARGs found in clinical (Fig. 4B) and pre-clinical (Fig. 4C) class 1 integron sequences was found in freshwater communities exposed to antibiotics. None of the pollutants had a significant impact on overall ARG detection in class 1 integrons. In addition, the abundance of class 1 integrons determined by qPCR increased very slightly in the presence of antibiotics, microplastics and both combined (Fig. 5A). Despite the variable increase of the abundance of the integron-associated gentamicin resistance gene *aac(6’)-II* and the ciprofloxacin resistance gene *qnrB* in the non-antibiotic exposed plastisphere, no effects (at *P* < 0.05) were observed in the presence of pollution. Only the abundance of the *aac(6’)-Ib-cr,* detected in clinical class 1 integron sequences and related to ciprofloxacin resistance increased under antibiotic exposure, both in freshwater and in the plastisphere. None of the genes involved in gentamicin and ciprofloxacin resistance detected in the metagenomic analyses (including those quantified by qPCR) were found in plasmid contigs (Table S6 in Supplementary Information).

**Fig. 6:**
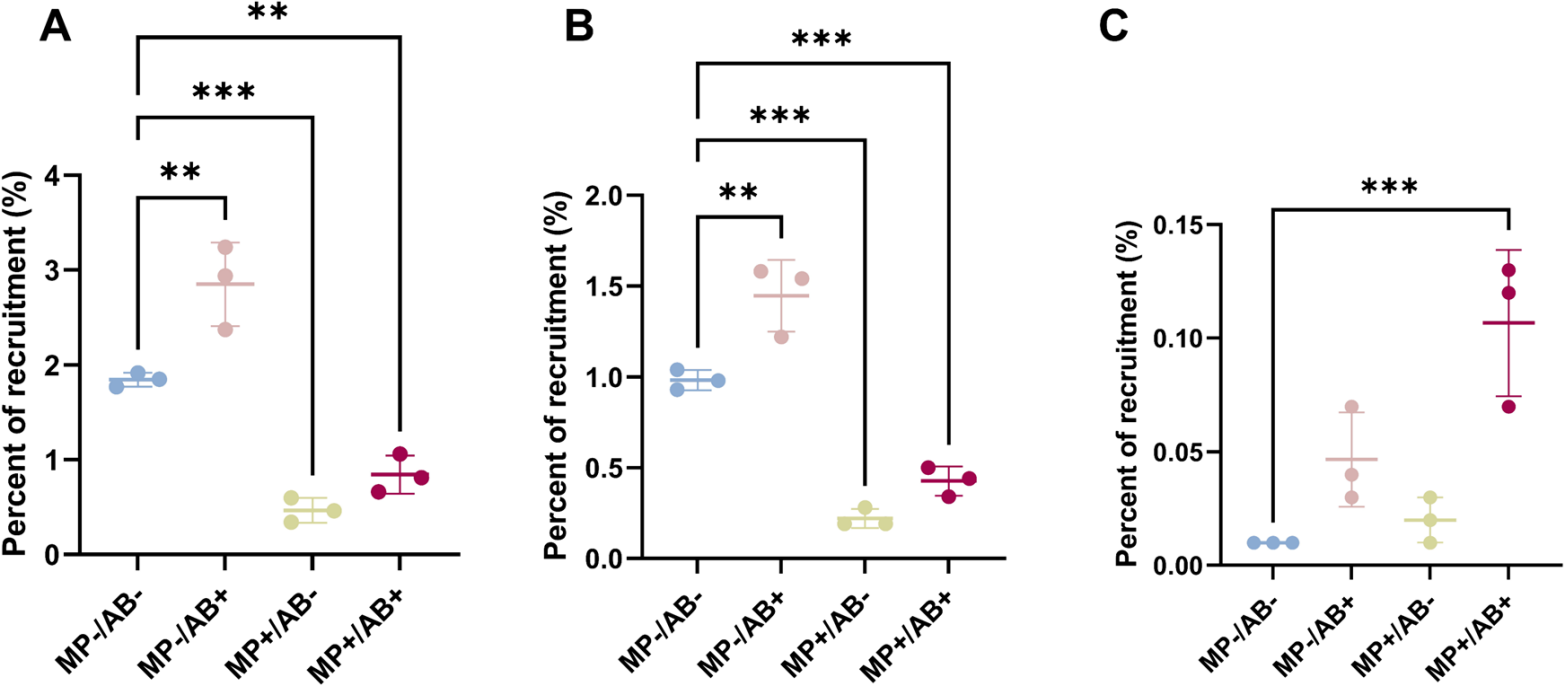
Relative abundance of the ARG-containing viral contigs obtained from the viral assembly of short metagenomic reads. **A** Viral contig 1. **B** Viral contig 2. **C** Viral contig 3. The percent of recruitment represents the percentage of reads from a sample that map onto each contig and is thus normalized by sequencing depth. ANOVA *p*-values: < 0.0001 (**A**, **B**); 0.0014 (**C**). Only pairwise comparisons with *p*-value < 0.05 are shown. *n* = 3

More ARG-containing viral contigs were obtained from the short reads than from the hybrid assembly of short and long reads (Table S7 in Supplementary Information). Thus, further analyses were done on the co-assembly of all short reads together. Three viral contigs belonging to the *Caudoviricetes* bacteriophage class and containing ARGs were obtained from the co-assembly of all short reads together (Fig. 6). Viral contigs 1 and 2 (Fig. 6A,B) showed an increased relative abundance in antibiotic-exposed freshwater and a reduced abundance in the plastisphere. VC1 contained a L1 β-lactamase and its putative host was *Pseudomonas aeruginosa* (Table 2)*.* VC2 contained a *aph(3’)-IIc* gentamicin resistance gene and its putative host was *Stenotrophomonas maltophilia* (Table 2)*.* Both antibiotic resistance genes were found in the *Stenotrophomonas maltophilia* complete MAG, and when this MAG was aligned to the viral contigs, both were found within the *Stenotrophomonas maltophilia* genome (Table S8 in Supplementary Information). The MAG aligned to VC1 had a 100% identity over the whole viral contig length and to VC2 at a 99.995% identity over 115,208 of the 115,306 nucleotides of the viral contig. This suggests the presence of two prophages in the *Stenotrophomonas maltophilia* genome. Finally, viral contig 3 increased its relative abundance under antibiotic pollution, particularly in the plastisphere (Fig. 6C). It contained an *axyXY* gene related to ciprofloxacin and gentamicin resistance, its putative host was *Sinorhizobium meliloti* (Table 2) and it was found in the MAG associated to *Achromobacter (99*.8% identity over 24,835 of the 38,066 nucleotides of the viral contig).

**Table 2:** Taxonomy, content, putative hosts, and presence in MAGs of the ARG-containing viral contigs identified in this study

Viral contig ID	Contig length (bp)	ARGs	Taxonomy	Genes	Completeness	Host	MAGs containing the contig
VC1	232,259	L1* (β-lactams)	*Caudoviricetes*	203	ND	*P. aeruginosa*	*S. maltophilia*
VC2	115,306	aph(3’)-IIc* (gentamicin)	*Caudoviricetes*	111	Medium quality	*S. maltophilia*	*S. maltophilia*
VC3	38,066	axyXY** (CPX + GM)	*Caudoviricetes*	31	ND	*Sinorhizobium meliloti*	*Achromobacter*

*VC* viral contig.

*L1 and aph(3’)-IIc were also found in the Stenotrophomonas maltophilia MAG.

**AxyXY was also found in the *Achromobacter* MAG. Viral contig taxonomy, genetic content, completeness, and putative hosts were determined by PhageScope. To determine if the viral contigs were contained in MAG sequences, a nucleotide blast analysis at > 99% similarity and > 1000 alignment length was performed.

## Discussion

The ubiquitous presence of microplastics in surface waters may induce changes in antibiotic selection behavior and in the structure and functioning of aquatic bacterial communities due to the selective nature of the plastisphere (microorganisms attached to the plastic). The selective impact of microplastics on the microbial communities present in aquatic ecosystems could lead to changes in their antibiotic resistome [29] and could have consequences in clinics. The main goal of this study was to explore the microbial ecology of the antibiotic-exposed plastisphere microbiome and determine how the response of the plastisphere communities to antibiotics at sub-lethal levels compares to that of freshwater ecosystems. Our research shows how the plastisphere selective environment influences bacterial dynamics and induces a shift in the response to antibiotics.

Antibiotics at sub-lethal doses arguably have the strongest impact on freshwater bacteria. Bacterial communities exposed to ciprofloxacin and gentamicin at sub-lethal levels had the most distinct composition. Some of the ASVs that showed an increased abundance in the 16S rRNA gene sequences upon antibiotic exposure (Fig. 2A) are associated to genera (*Herbaspirillum, Stenotrophomonas)* that contained both ciprofloxacin and gentamicin resistance genes in the MAGs obtained in this study. Besides, these MAGs were also enriched in the antibiotic-exposed freshwater. While ciprofloxacin resistance genes were found in 100% of the complete reference genomes of *Herbaspirillum* and *Stenotrophomonas* available at NCBI (Table S9 in Supplementary Information), suggesting a possible intrinsic resistance to this antibiotic, no gentamicin resistance genes were found in these reference genomes. Our results show that a gentamicin resistance gene present in the genome of *Stenotrophomonas maltophilia* is encoded by a prophage integrated into its genome, suggesting that this putative ARB could have acquired this resistance through transduction (Table 2). *Stenotrophomonas maltophilia* is an opportunistic pathogen with a wide range of resistance mechanisms and is considered a serious threat to human health [74]. Moreover, another putative ARB related to the genus *Achromobacter* that increased its abundance under antibiotic sub-lethal pressure in freshwater was identified in this study using assembly-based metagenomic approaches. This MAG also contained genetic mechanisms to resist ciprofloxacin and gentamicin pressure as did the associated reference genomes found in the NCBI database. All these putative ARB contained virulence genes and genes related to conjugation. In addition, antibiotic exposure induced the selection of a ciprofloxacin resistance gene, *aac(6’)-Ib-cr*, an increase in class 1 integron abundance and in the proportion of ARGs carried by these integrons. This latter effect relates to changes in the integron-associated resistome, and it could be a consequence of the selection of integrons and/or integron-carrying bacteria or of gene mobilization into class 1 integrons. These findings are consistent with previous studies from our group showing the impact of antibiotic sub-inhibitory pressure on bacterial community structure, ARB and ARG selection, and mobilization in class 1 integrons [15, 16].

Nevertheless, the presence of microplastics also had a selective effect on freshwater bacteria. We observed a selection of specific members of the community, such as *Acinetobacter.* ASV related to this genus showed an increased abundance in the plastisphere (Fig. 2) and the same trend was observed in the ARG-containing MAG associated to this genus (Fig. 3C). *Acinetobacter* is a well-known biofilm member [75] commonly found in the plastisphere [30, 35, 40]. *Aeromonas* was also present containing virulence genes and ARGs conferring resistance to a wide range of antibiotics and is a genus associated with pathogenicity in humans [76]. All of the available complete reference genomes of *Acinetobacter* and *Aeromonas* contained ciprofloxacin efflux mechanisms (Figure S9 in Supplementary Information), similarly to the results observed in our MAGs, suggesting an intrinsic resistance to sub-lethal levels of ciprofloxacin in these genomes. None of these MAGs contained plastic degradation genes, which suggests their success in the plastisphere could be related to their ability to form biofilms on the plastic surface rather than to plastic degradation mechanisms. The selective nature of the plastisphere favoring bacteria that can colonize onto the plastic surface has been extensively reported in the past [77] and is supported by the lower biomass observed in the non-antibiotic-exposed plastisphere and the lower overall ARG load detected in the plastisphere. On the other hand, a significant increase of bacterial biomass was observed in the plastisphere exposed to antibiotics (Fig. 1) without a concomitant shift in bacterial community composition. Antibiotic exposure might favor the development of the biofilm in the plastisphere. A study where *E. coli* was exposed to a combination of polystyrene, the microplastic type used in this study, and norfloxacin, a fluoroquinolone, showed a stimulatory effect on growth induced by the mixture of these two pollutants [78]. In addition, both ciprofloxacin and gentamicin at sub-inhibitory concentrations have been associated to a stimulation of biofilm formation [79, 80]. These mechanisms could underly the increase in bacterial biomass in the antibiotic-exposed plastisphere and further research should explore this matter.

Finally, sub-lethal concentrations of antibiotics had a different effect on bacterial communities in freshwater and in the plastisphere. Bacteria that are able to colonize the plastisphere showed a more stable community composition than river water communities under antibiotic exposure (Fig. 2, Figure S3) and an overall reduced response to antibiotic pressure at the genetic level with the sole exception of the increased abundance of the ciprofloxacin resistance gene *aac(6’)-Ib-cr* (Fig. 5D). This stability in the community structure in the plastisphere is consistent to the extensively-described increased tolerance of biofilms to antibiotics [81, 82]. In addition, the presence of different ASVs of *Acinetobacter, Chryseobacterium,* and *Stenotrophomonas* that changed in abundance under antibiotic, microplastic, and combined pressure indicates that bacterial subpopulations may respond differently to anthropogenic selective pressures and thus affect the outcome of environmental antibiotic resistance. The assembly-based analyses of metagenomic reads showed an enrichment of *Achromobacter* in the antibiotic-exposed plastisphere (Fig. 3A), a genus associated to respiratory tract infections and intrinsically resistant to several antibiotics [83]. Although the relative abundance of other community members, such as *Herbaspirillum* (Fig. 3B)*,* a genus associated to rare infections in humans [84], and *Stenotrophomonas maltophilia* (Fig. 3C)*,* a well-known opportunistic pathogen, were significantly reduced in the plastisphere, they increased under antibiotic exposure in the plastisphere. Furthermore, three MAGs associated to *Comamonas, Chryseobacterium,* and an unknown *Enterobacteriaceae* were significantly enriched in the antibiotic-exposed plastisphere (Fig. 3F–H). The presence of ciprofloxacin resistance mechanisms in the *Comamonas* MAG and of ciprofloxacin and gentamicin resistance mechanisms in the MAG associated to an *Enterobacteriaceae* could partially explain their success in the antibiotic-exposed plastisphere, although there is some variability in the presence of resistance mechanisms to both antibiotics in the reference genomes of *Comamonas* (Table S9 in Supplementary Information). Nevertheless, the absence of resistance mechanisms to any of these antibiotics in both the *Chryseobacterium* MAG (Table 1) and complete reference genomes (Table S9 in Supplementary Information) raises questions about its enrichment in the antibiotic-exposed plastisphere. Besides, *Chryseobacterium* ASVs showed different behavior under antibiotic, microplastic, or combined pressures (Fig. 2). Our results suggest that other mechanisms could favor the survival of some *Chryseobacterium* populations in the antibiotic-exposed plastisphere even though they do not contain any antibiotic resistance mechanisms. Some potential mechanisms underlying this phenomenon could involve biofilm formation, persistence, unspecific stress responses, or the presence of antibiotic resistance genes in plasmids that were overlooked in this study. Although the evaluation of these mechanisms was out of the scope of this study, further research should shed some light into the enrichment of (allegedly) non-antibiotic-resistant members in the antibiotic-exposed plastisphere.

All of the MAGs maintained in the antibiotic-exposed plastisphere except *Chryseobacterium* contained some ARGs associated to class 1 integrons and genes related to horizontal gene transfer. In addition to the potential of dissemination of these ARGs to other bacterial hosts in the biofilm, the presence of virulence genes in these MAGs raises concerns about their pathogenicity to humans. For example, the presence in the plastisphere of potentially mobilizable ARGs and virulence genes in *Stenotrophomonas maltophilia* could be considered a high risk for human health [85]. Thus, microplastics could also contribute to the maintenance of the selected communities through biofilm formation and increased tolerance to antibiotic exposure and other environmental perturbations.

In addition, although no ciprofloxacin nor gentamicin resistance genes were found in plasmid contigs, the assembly of viral contigs enabled the identification of three ARG-containing contigs (Table 2). One of these contained a gentamicin resistance gene, *aph(3’)-IIc,* that was identified in the complete MAG of *Stenotrophomonas maltophilia.* Besides, the putative host of this viral contig was *Stenotrophomonas maltophilia,* and the bacteriophage sequence was found within its genome, suggesting the integration of the bacteriophage genetic content in a prophage form. Another potential transduction event involving both ciprofloxacin and gentamicin resistance was identified in the MAG of *Achromobacter* with a *axyXY-*containing bacteriophage integrated in its genome. Our results identify two potential transduction events involving gentamicin and ciprofloxacin resistance genes that could have led to the acquisition of new resistance mechanisms by two opportunistic pathogens, *Stenotrophomonas maltophilia* and *Achromobacter.* This research points to a possible underestimation of the role of transduction in environmental settings and underlines the need to include bacteriophages in future environmental resistome and mobilome studies. Furthermore, the absence of antibiotic resistance genes in plasmid contigs could imply an overestimation of the role that conjugation plays in environmental settings, where the contact between bacteria is reduced compared to in vitro scenarios and plasmids (or the ARGs they carry) might be lost in the absence of strong selective pressures. However, the lack of detection of ARGs in plasmid contigs could also reflect a methodological flaw in the obtention, sequencing and/or assembly of these mobile genetic elements. Further research should explore the potential biases associated to the study of the plasmidome to address these questions.

Finally, although it was not the main purpose of this study, our results reflect the biases associated to metagenomic approaches. For instance, genome assembly approaches performed differently, with OPERA-MS overall being superior to the assembly of short reads using MEGAHIT and the hybrid assembly of short and long reads using Unicycler. This was not surprising, since hybrid assemblies optimized for metagenomic datasets such as OPERA-MS [86] usually outperform short-read approaches, and Unicycler is less adapted to complex datasets and shows an optimal performance with pathogen genomes [87]. However, some information was missing from the OPERA-MS hybrid assembly. *Acinetobacter* contigs could not be binned into a MAG of sufficient quality, whereas a 78% completion, 2% redundancy *Acinetobacter* MAG was obtained from short reads, and the *Stenotrophomonas maltophilia* MAG obtained using OPERA-MS showed the highest redundancy (7%) of the three approaches, whereas a complete MAG with 0% redundancy was observed using Unicycler. These differences support previous conclusions about potential misassembly between similar sequences using hybrid assembly approaches [88]. Another example of methodological biases is the underestimation for class 1 integron sequences in the metagenomic reads. No integrons were found in non-assembled reads and in the hybrid assemblies, whereas only five integrons were identified in non-binned contigs from the short read assembly and none in the MAGs (results not shown). However, class 1 integrons were detected by qPCR and sequenced, and they contained ARGs that were relevant to this study. Finally, as mentioned above, the used of total (chromosomal and hopefully plasmid) DNA might reduce the sensitivity of plasmid DNA detection in sequencing approaches and result in misleading conclusions about the environmental mobilome. This could lead to an underestimation of the role of the plasmidome and potential transformation, conjugation or vesiculation events taking place in the environment. These aspects should be carefully considered in the future, and research efforts should include a variety of DNA extracts (bacterial, viral, plasmid DNA), sequencing targets (metagenomes, plasmids, bacteriophages, integrons, specific genes), long and short read platforms and several analysis approaches (non-assembled read screening and multiple assembly approaches) to obtain a full picture of the environmental resistome and the potential risks it poses to human health.

Considering the low degradability of microplastics [21] and their potential role as a vector capable of protecting bacteria from environmental stressors, transporting them over a long transport range and disseminating them to surrounding environments through detachment from the plastisphere [30], the risk associated to their presence goes beyond simply an enrichment of ARB and ARGs in the plastisphere. Many antibiotics are easily degraded in the environment and the resistance mechanisms they select for could be loss from the communities in the absence of selective pressure. Yet, the maintenance of these resistance mechanisms in the plastisphere alone implies a potential risk for human health. In order to quantify the risk of antibiotic resistance dissemination associated to surface water microplastic pollution, future efforts should focus on long-term studies evaluating the persistence of ARB and ARGs in the plastisphere, the evolution of the microplastic structure and its behavior in a complex scenario with interactions with other pollutants [46, 89], and other environmental stresses such as temperature [90] or UV exposure [91].

## Conclusions

This research shows how the selective nature of the plastisphere changes the response of bacterial communities to antibiotic pressure at sub-lethal doses. Here we identify microbial responses that improve our understanding on the selective role of the plastisphere and its impact on the maintenance of environmental antibiotic resistance in combination with other anthropogenic pollutants. Our research identified bacteriophages as potential key players on the dissemination of antibiotic resistance in environmental settings. This work provides new insights into the microbial ecology of the antibiotic-exposed plastisphere and highlights the need to evaluate the impact of aquatic pollutants in environmental communities using complex scenarios with combined stresses.

### Supplementary Information

**Additional File 1. **Table with ASV abundance and taxonomic annotation.

**Additional File 2. **ASV sequences.

**Additional File 3.**

## Data Availability

The datasets generated and analyzed during the current study are available at the DDBJ repository, BioProject PRJDB17794, DRA accession DRA018264. The code used to analyze all the sequences included in this study is available at: https://github.com/concscid/Joannard-and-Sanchez-Cid-2024

## References

[1] Sommer MOA, Munck C, Toft-Kehler RV, Andersson DI (2017). Prediction of antibiotic resistance: time for a new preclinical paradigm?. Nat Rev Microbiol.

[2] Levy SB, Bonnie M (2004). Antibacterial resistance worldwide: causes, challenges and responses. Nat Med.

[3] van Hengel AJ, Marin L (2019). Research, innovation, and policy: an alliance combating antimicrobial resistance. Trends Microbiol..

[4] Zhu G, Wang X, Yang T, Su J, Qin Y, Wang S (2020). Air pollution could drive global dissemination of antibiotic resistance genes. ISME J.

[5] Karkman A, Pärnänen K, Larsson DGJ. Fecal pollution explains antibiotic resistance gene abundances in anthropogenically impacted environments. Nat Commun. 2019;10(80). 10.1038/s41467-018-07992-3. 10.1038/s41467-018-07992-3 PMC6325112 30622259

[6] Gaze WH, Zhang L, Abdouslam NA, Hawkey PM, Calvo-Bado L, Royle J (2011). Impacts of anthropogenic activity on the ecology of class 1 integrons and integron-associated genes in the environment. ISME J.

[7] Wellington EMH, Boxall ABA, Cross P, Feil EJ, Gaze WH, Hawkey PM (2013). The role of the natural environment in the emergence of antibiotic resistance in Gram-negative bacteria. Lancet Infect Dis.

[8] Zhang QQ, Ying GG, Pan CG, Liu YS, Zhao JL (2015). Comprehensive evaluation of antibiotics emission and fate in the river basins of China: source analysis, multimedia modeling, and linkage to bacterial resistance. Environ Sci Technol.

[9] Cairns J, Ruokolainen L, Hultman J, Tamminen M, Virta M, Hiltunen T (2018). Ecology determines how low antibiotic concentration impacts community composition and horizontal transfer of resistance genes. Commun Biol.

[10] Murray AK, Zhang L, Yin X, Zhang T, Buckling A, Snape J (2018). Novel insights into selection for antibiotic resistance in complex microbial communities. mBio..

[11] Resistance NA, Medicine H, Farming A, Lau CHF, van Engelen K, Gordon S (2017). Novel antibiotic resistance determinants from agricultural soil exposed to antibiotics widely used in human medicine and animal farming. Appl Environ Microbiol..

[12] Elder FCT, Proctor K, Barden R, Gaze WH, Snape J, Feil EJ (2021). Spatiotemporal profiling of antibiotics and resistance genes in a river catchment: human population as the main driver of antibiotic and antibiotic resistance gene presence in the environment. Water Res..

[13] Zhou LJ, Ying GG, Liu S, Zhao JL, Yang B, Chen ZF (2013). Occurrence and fate of eleven classes of antibiotics in two typical wastewater treatment plants in South China. Sci Total Environ.

[14] Andersson DI, Hughes D (2014). Microbiological effects of sublethal levels of antibiotics. Nat Rev Microbiol.

[15] Sanchez-Cid C, Guironnet A, Keuschnig C, Wiest L, Vulliet E, Vogel TM. Gentamicin at sub-inhibitory concentrations selects for antibiotic resistance in the environment. ISME Communications. 2022;2(29). 10.1038/s43705-022-00101-y. 10.1038/s43705-022-00101-y PMC9723587 37938295

[16] Sanchez-Cid C, Ghaly TM, Gillings MR, Vogel TM. Sub-inhibitory gentamicin pollution induces gentamicin resistance gene integration in class 1 integrons in the environment. Sci Rep. 2023;13(8612). 10.1038/s41598-023-35074-y. 10.1038/s41598-023-35074-y PMC10224954 37244902

[17] Wang Y, Lu J, Zhang S, Li J, Mao L, Yuan Z (2021). Non-antibiotic pharmaceuticals promote the transmission of multidrug resistance plasmids through intra- and intergenera conjugation. ISME J.

[18] Baker-Austin C, Wright MS, Stepanauskas R, McArthur JV (2006). Co-selection of antibiotic and metal resistance. Trends Microbiol.

[19] Pal C, Bengtsson-Palme J, Kristiansson E, Larsson DGJ. Co-occurrence of resistance genes to antibiotics, biocides and metals reveals novel insights into their co-selection potential. BMC Genomics. 2015;16(964). 10.1186/s12864-015-2153-5. 10.1186/s12864-015-2153-5 PMC4650350 26576951

[20] Zambrano MM (2023). Interplay between antimicrobial resistance and global environmental change.

[21] Wang Y, Yang Y, Liu X, Zhao J, Liu R, Xing B (2021). Interaction of microplastics with antibiotics in aquatic environment: distribution, adsorption, and toxicity. Environ Sci Technol.

[22] Thompson RC, Olsen Y, Mitchell RP, Davis A, Rowland SJ, John AWG (1979). Lost at sea: where is all the plastic?. Science.

[23] Mitrano DM, Wohlleben W. Microplastic regulation should be more precise to incentivize both innovation and environmental safety. Nat Commun. 2020;11(5324). 10.1038/s41467-020-19069-1. 10.1038/s41467-020-19069-1 PMC7578016 33087714

[24] Dusaucy J, Gateuille D, Perrette Y, Naffrechoux E. Microplastic pollution of worldwide lakes. Environ Pollut. 2021;284(117075). 10.1016/j.envpol.2021.117075. 10.1016/j.envpol.2021.117075 33894537

[25] Bhatt V, Chauhan JS (2023). Microplastic in freshwater ecosystem: bioaccumulation, trophic transfer, and biomagnification. Environ Sci Pollut Res.

[26] Xu S, Ma J, Ji R, Pan K, Miao AJ. Microplastics in aquatic environments: occurrence, accumulation, and biological effects. Sci Total Environ. 2020;703(134699). 10.1016/j.scitotenv.2019.134699. 10.1016/j.scitotenv.2019.134699 31726297

[27] Zettler ER, Mincer TJ, Amaral-Zettler LA (2013). Life in the “plastisphere”: Microbial communities on plastic marine debris. Environ Sci Technol.

[28] Wang J, Qin X, Guo J, Jia W, Wang Q, Zhang M, et al. Evidence of selective enrichment of bacterial assemblages and antibiotic resistant genes by microplastics in urban rivers. Water Res. 2020;183(116113). 10.1016/j.watres.2020.116113. 10.1016/j.watres.2020.116113 32668354

[29] Luo G, Liang B, Cui H, Kang Y, Zhou X, Tao Y, et al. Determining the contribution of micro/nanoplastics to antimicrobial resistance: challenges and perspectives. Environ Sci Technol. 2023;57(33):12137–52. 10.1021/acs.est.3c01128. 10.1021/acs.est.3c01128 37578142

[30] Silva I, Rodrigues ET, Tacão M, Henriques I. Microplastics accumulate priority antibiotic-resistant pathogens: evidence from the riverine plastisphere. Environ Pollut. 2023;332(121995). 10.1016/j.envpol.2023.121995. 10.1016/j.envpol.2023.121995 37302790

[31] Li R, Zhu L, Wang Y, Zhu YG. Metagenomic insights into environmental risk of field microplastics in an urban river. Water Res. 2022;223(119018). 10.1016/j.watres.2022.119018. 10.1016/j.watres.2022.119018 36057234

[32] Luo T, Dai X, Wei W, Xu Q, Ni B-J (2023). Microplastics enhance the prevalence of antibiotic resistance genes in anaerobic sludge digestion by enriching antibiotic-resistant bacteria in surface biofilm and facilitating the vertical and horizontal gene transfer. Environ Sci Technol.

[33] Arias-Andres M, Klümper U, Rojas-Jimenez K, Grossart HP (2018). Microplastic pollution increases gene exchange in aquatic ecosystems. Environ Pollut.

[34] Li LG, Zhang T. Plasmid-mediated antibiotic resistance gene transfer under environmental stresses: insights from laboratory-based studies. Sci Total Environ. 2023;887(163870). 10.1016/j.scitotenv.2023.163870. 10.1016/j.scitotenv.2023.163870 37149187

[35] Luo T, Dai X, Chen Z, Wu L, Wei W, Xu Q, et al. Different microplastics distinctively enriched the antibiotic resistance genes in anaerobic sludge digestion through shifting specific hosts and promoting horizontal gene flow. Water Res. 2023;228(119356). 10.1016/j.watres.2022.119356. 10.1016/j.watres.2022.119356 36423550

[36] Chan SY, Liu SY, Wu R, Wei W, Fang JKH, Chua SL (2023). simultaneous dissemination of nanoplastics and antibiotic resistance by nematode couriers. Environ Sci Technol.

[37] Yang Y, Zhang X, Jiang J, Han J, Li W, Li X (2022). Which micropollutants in water environments deserve more attention globally?. Environ Sci Technol.

[38] Liu Y, Liu W, Yang X, Wang J, Lin H, Yang Y. Microplastics are a hotspot for antibiotic resistance genes: progress and perspective. Sci Total Environ. 2021;773(145643). 10.1016/j.scitotenv.2021.145643. 10.1016/j.scitotenv.2021.145643 33940744

[39] Song X, Zhuang W, Cui H, Liu M, Gao T, Li A, et al. Interactions of microplastics with organic, inorganic and bio-pollutants and the ecotoxicological effects on terrestrial and aquatic organisms. Sci Total Environ. 2022;838(156068). 10.1016/j.scitotenv.2022.156068. 10.1016/j.scitotenv.2022.156068 35598660

[40] Zheng Z, Huang Y, Liu L, Wang L, Tang J. Interaction between microplastic biofilm formation and antibiotics: effect of microplastic biofilm and its driving mechanisms on antibiotic resistance gene. J Hazard Mater. 2023;459(132099). 10.1016/j.jhazmat.2023.132099. 10.1016/j.jhazmat.2023.132099 37517232

[41] Liu X, Wang H, Li L, Deng C, Chen Y, Ding H, et al. Do microplastic biofilms promote the evolution and co-selection of antibiotic and metal resistance genes and their associations with bacterial communities under antibiotic and metal pressures? J Hazard Mater. 2022;424(127285). 10.1016/j.jhazmat.2021.127285. 10.1016/j.jhazmat.2021.127285 34597934

[42] Wang S, Xue N, Li W, Zhang D, Pan X, Luo Y. Selectively enrichment of antibiotics and ARGs by microplastics in river, estuary and marine waters. Sci Total Environ. 2020;708(134594). 10.1016/j.scitotenv.2019.134594. 10.1016/j.scitotenv.2019.134594 31796269

[43] Wang Z, Gao J, Zhao Y, Dai H, Jia J, Zhang D. Plastisphere enrich antibiotic resistance genes and potential pathogenic bacteria in sewage with pharmaceuticals. Sci Total Environ. 2021;768(144663). 10.1016/j.scitotenv.2020.144663. 10.1016/j.scitotenv.2020.144663 33454495

[44] Yang K, Chen QL, Chen ML, Li HZ, Liao H, Pu Q (2020). Temporal dynamics of antibiotic resistome in the plastisphere during microbial colonization. Environ Sci Technol.

[45] Wang J, Peng C, Dai Y, Li Y, Jiao S, Ma X, et al. Slower antibiotics degradation and higher resistance genes enrichment in plastisphere. Water Res. 2022;222(118920). 10.1016/j.watres.2022.118920. 10.1016/j.watres.2022.118920 35964510

[46] Niegowska M, Sanseverino I, Navarro A, Lettieri T (2021). Knowledge gaps in the assessment of antimicrobial resistance in surface waters. FEMS Microbiol Ecol.

[47] Le-minh N, Khan SJ, Drewes JE, Stuetz RM (2010). Fate of antibiotics during municipal water recycling treatment processes. Water Res.

[48] Shun-Mei E, Zeng JM, Yuan H, Lu Y, Cai RX, Chen C (2017). Sub-inhibitory concentrations of fluoroquinolones increase conjugation frequency. Microb Pathog.

[49] Watanabe K, Kodama Y, Harayama S (2001). Design and evaluation of PCR primers to amplify bacterial 16S ribosomal DNA fragments used for community fingerprinting. J Microbiol Methods.

[50] Walters W, Hyde ER, Berg-Lyons D, Ackermann G, Humphrey G, Parada A, et al. Improved bacterial 16S rRNA gene (V4 and V4–5) and fungal internal transcribed spacer marker gene primers for microbial community surveys. mSystems. 2016;1(10.1128). 10.1128/msystems.00009-15. 10.1128/mSystems.00009-15 PMC5069754 27822518

[51] Callahan BJ, McMurdie PJ, Rosen MJ, Han AW, Johnson AJA, Holmes SP (2016). DADA2: high resolution sample inference from Illumina amplicon data. Nat Methods.

[52] Cole JR, Wang Q, Fish JA, Chai B, McGarrell DM, Sun Y (2014). Ribosomal Database Project: data and tools for high throughput rRNA analysis. Nucleic Acids Res.

[53] Love MI, Huber W, Anders S. Moderated estimation of fold change and dispersion for RNA-seq data with DESeq2. Genome Biol. 2014;15(550). 10.1186/s13059-014-0550-8. 10.1186/s13059-014-0550-8 PMC4302049 25516281

[54] Minoche AE, Dohm JC, Himmelbauer H. Evaluation of genomic high-throughput sequencing data generated on Illumina HiSeq and Genome Analyzer systems. Genome Biol. 2011;12(R112). 10.1186/gb-2011-12-11-r112. 10.1186/gb-2011-12-11-r112 PMC3334598 22067484

[55] Alcock BP, Raphenya AR, Lau TTY, Tsang KK, Bouchard M, Edalatmand A (2020). CARD 2020: antibiotic resistome surveillance with the comprehensive antibiotic resistance database. Nucleic Acids Res.

[56] Buchfink B, Xie C, Huson DH (2015). Fast and sensitive protein alignment using DIAMOND. Nat Methods..

[57] Li D, Liu CM, Luo R, Sadakane K, Lam TW (2015). MEGAHIT: an ultra-fast single-node solution for large and complex metagenomics assembly via succinct de Bruijn graph. Bioinformatics.

[58] Langmead B, Salzberg SL (2012). Fast gapped-read alignment with Bowtie 2. Nat Methods.

[59] Eren AM, Esen OC, Quince C, Vineis JH, Morrison HG, Sogin ML (2015). Anvi’o: an advanced analysis and visualization platformfor ’omics data. PeerJ.

[60] Bowers RM, Kyrpides NC, Stepanauskas R, Harmon-Smith M, Doud D, Reddy TBK (2017). Minimum information about a single amplified genome (MISAG) and a metagenome-assembled genome (MIMAG) of bacteria and archaea. Nat Biotechnol.

[61] Wick RR, Judd LM, Gorrie CL, Holt KE (2017). Unicycler: Resolving bacterial genome assemblies from short and long sequencing reads. PLoS Comput Biol.

[62] Bertrand D, Shaw J, Kalathiyappan M, Ng AHQ, Kumar MS, Li C (2019). Hybrid metagenomic assembly enables high-resolution analysis of resistance determinants and mobile elements in human microbiomes. Nat Biotechnol.

[63] Olson RD, Assaf R, Brettin T, Conrad N, Cucinell C, Davis JJ (2023). Introducing the Bacterial and Viral Bioinformatics Resource Center (BV-BRC): a resource combining PATRIC. IRD and ViPR. Nucleic Acids Res..

[64] Gambarini V, Pantos O, Kingsbury JM, Weaver L, Handley KM, Lear G. PlasticDB: a database of microorganisms and proteins linked to plastic biodegradation. Database. 2022;2022:baac008. 10.1093/database/baac008. 10.1093/database/baac008 PMC9216477 35266524

[65] Yu MK, Fogarty EC, Eren AM. The genetic and ecological landscape of plasmids in the human gut. bioRxiv. 2023;2020.11.01.361691. 10.1101/2020.11.01.361691.

[66] Antipov D, Raiko M, Lapidus A, Pevzner PA (2019). Plasmid detection and assembly in genomic and metagenomic data sets. Genome Res.

[67] Antipov D, Raiko M, Lapidus A, Pevzner PA (2020). Metaviral SPAdes: assembly of viruses from metagenomic data. Bioinformatics.

[68] Wang RH, Yang S, Liu Z, Zhang Y, Wang X, Xu Z (2024). PhageScope: a well-annotated bacteriophage database with automatic analyses and visualizations. Nucleic Acids Res.

[69] Holmes AJ, Gillings MR, Nield BS, Mabbutt BC, Nevalainen KMH, Stokes HW (2003). The gene cassette metagenome is a basic resource for bacterial genome evolution. Environ Microbiol.

[70] Gillings MR, Xuejun D, Hardwick SA, Holley MP, Stokes HW (2009). Gene cassettes encoding resistance to quaternary ammonium compounds: a role in the origin of clinical class 1 integrons?. ISME J.

[71] Park CH, Robicsek A, Jacoby GA, Sahm D, Hooper DC (2006). Prevalence in the United States of aac(6′)-Ib-cr encoding a ciprofloxacin-modifying enzyme. Antimicrob Agents Chemother.

[72] Rutgersson C, Fick J, Marathe N, Kristiansson E, Janzon A, Angelin M (2014). Fluoroquinolones and qnr genes in sediment, water, soil, and human fecal flora in an environment polluted by manufacturing discharges. Environ Sci Technol.

[73] Wang FH, Qiao M, Su JQ, Chen Z, Zhou X, Zhu YG (2014). High throughput profiling of antibiotic resistance genes in urban park soils with reclaimed water irrigation. Environ Sci Technol.

[74] Brooke JS (2012). Stenotrophomonas maltophilia: an emerging global opportunistic pathogen. Clin Microbiol Rev.

[75] McConnell MJ, Actis L, Pachón J (2013). Acinetobacter baumannii: human infections, factors contributing to pathogenesis and animal models. FEMS Microbiol Rev.

[76] Janda JM, Abbott SL (2010). The genus Aeromonas: taxonomy, pathogenicity, and infection. Clin Microbiol Rev.

[77] Entezari S, Al MA, Mostashari A, Ganjidoust H, Ayati B, Yang J. Microplastics in urban waters and its effects on microbial communities: a critical review. Environ Sci Pollut Res. 2022;29(59):88410–31. 10.1007/s11356-022-23810-2 36327084

[78] Shen H, Yang M, Yin K, Wang J, Tang L, Lei B, et al. Size- and surface charge-dependent hormetic effects of microplastics on bacterial resistance and their interactive effects with quinolone antibiotic. Sci Total Environ. 2023;903(166580). 10.1016/j.scitotenv.2023.166580. 10.1016/j.scitotenv.2023.166580 37633387

[79] George J, Halami PM (2017). Sub-inhibitory concentrations of gentamicin triggers the expression of aac(6′)Ie-aph(2″)Ia, chaperons and biofilm related genes in Lactobacillus plantarum MCC 3011. Res Microbiol.

[80] Morita Y, Tomida J, Kawamura Y. Responses of Pseudomonas aeruginosa to antimicrobials. Front Microbiol. 2013;4(422). 10.3389/fmicb.2013.00422. 10.3389/fmicb.2013.00422 PMC3884212 24409175

[81] Ciofu O, Moser C, Jensen PØ, Høiby N (2022). Tolerance and resistance of microbial biofilms. Nat Rev Microbiol.

[82] Hall CW, Mah TF (2017). Molecular mechanisms of biofilm-based antibiotic resistance and tolerance in pathogenic bacteria. FEMS Microbiol Rev.

[83] Isler B, Kidd TJ, Stewart AG, Harris P, Paterson DL. Achromobacter infections and treatment options. Antimicrob Agents Chemother. 2020;64:10.1128/aac.01025-20. 10.1128/aac.01025-20. 10.1128/AAC.01025-20 PMC7577122 32816734

[84] Bloise I, Guedez-López GV, Tejedor-Rodríguez M, Romero-Gómez MP, García-Rodríguez J, Mingorance J (2021). Bloodstream infection due to Herbaspirillum sp.: case series and review of literature. Eur J Clin Microbiol Infect Dis..

[85] Martínez JL, Coque TM, Baquero F (2015). What is a resistance gene? Ranking risk in resistomes. Nat Rev Microbiol.

[86] Brown CL, Keenum IM, Dai D, Zhang L, Vikesland PJ, Pruden A. Critical evaluation of short, long, and hybrid assembly for contextual analysis of antibiotic resistance genes in complex environmental metagenomes. Sci Rep. 2021;11(3753). 10.1038/s41598-021-83081-8. 10.1038/s41598-021-83081-8 PMC7881036 33580146

[87] Chen Z, Erickson DL, Meng J. Benchmarking hybrid assembly approaches for genomic analyses of bacterial pathogens using Illumina and Oxford Nanopore sequencing. BMC Genomics. 2020;21(631). 10.1186/s12864-020-07041-8. 10.1186/s12864-020-07041-8 PMC7490894 32928108

[88] Yorki S, Shea T, Cuomo CA, Walker BJ, LaRocque RC, Manson AL, et al. Comparison of long- and short-read metagenomic assembly for low-abundance species and resistance genes. Brief Bioinform. 2023;24. 10.1093/bib/bbad050. 10.1093/bib/bbad050 PMC10025444 36804804

[89] Stapleton MJ, Ansari AJ, Hai FI. Antibiotic sorption onto microplastics in water: a critical review of the factors, mechanisms and implications. Water Res. 2023;233(119790). 10.1016/j.watres.2023.119790. 10.1016/j.watres.2023.119790 36870107

[90] Zhu D, Ma J, Li G, Rillig MC, Zhu YG (2022). Soil plastispheres as hotpots of antibiotic resistance genes and potential pathogens. ISME J.

[91] Tian Y, Zhu J, Ying C, Luo H, Zhang S, Zhang L, et al. Photoaging processes of polyvinyl chloride microplastics enhance the adsorption of tetracycline and facilitate the formation of antibiotic resistance. Chemosphere. 2023;320(137820). 10.1016/j.chemosphere.2023.137820. 10.1016/j.chemosphere.2023.137820 36736841

