# Supplementary material for: Bacterial dynamics of the plastisphere microbiome exposed to sub-lethal antibiotic pollution
Source: Microbiome. 2024 May 24;12:97. doi: 10.1186/s40168-024-01803-2 (PMC11127405; doi:10.1186/s40168-024-01803-2)
Supplement: Supplementary file 4 — Additional File 3. [file 40168_2024_1803_MOESM3_ESM.docx]

**Supplementary information**

| **Sample** | **Input** | **Filtered** | **DenoisedF** | **DenoisedR** | **Merged** | **Non-chimeric** |
| --- | --- | --- | --- | --- | --- | --- |
| MP-/AB- 1 | 32049 | 30215 | 30060 | 30073 | 29126 | 26527 |
| MP-/AB- 2 | 16928 | 15413 | 15279 | 15323 | 14866 | 13902 |
| MP-/AB- 3 | 28738 | 27192 | 26957 | 26985 | 26069 | 23686 |
| MP-/AB+ 1 | 37197 | 35019 | 34802 | 34937 | 34041 | 31712 |
| MP-/AB+ 2 | 9380 | 8824 | 8751 | 8783 | 8469 | 7946 |
| MP-/AB+ 3 | 25131 | 23621 | 23495 | 23545 | 22859 | 21136 |
| MP+/AB- 1 | 27659 | 24544 | 24227 | 24330 | 22773 | 19624 |
| MP+/AB- 2 | 33300 | 29020 | 28694 | 28731 | 27066 | 22425 |
| MP+/AB- 3 | 32706 | 29092 | 28745 | 28876 | 27465 | 25271 |
| MP+/AB+ 1 | 36511 | 31130 | 30959 | 30968 | 30059 | 28848 |
| MP+/AB+ 2 | 88452 | 74955 | 73797 | 74336 | 65037 | 39986 |
| MP+/AB+ 3 | 37788 | 31938 | 31868 | 31886 | 31545 | 31532 |

Table S1. Number of 16S rRNA gene reads that went through each step of the DADA2 pipeline.

| **Condition** | **Biological replicate** | **16S rRNA gene** | **Clinical integrons** | **Pre-clinical integrons** | **Short-read metagenomics** | **Long-read metagenomics** |
| --- | --- | --- | --- | --- | --- | --- |
| MP-/AB- | 1 | 26460 | 574996 | 344874 | 836394 | 26907 |
|  | 2 | 13896 | 527004 | 361649 | 563312 | 21297 |
|  | 3 | 23589 | 507211 | 315295 | 298708 | 20922 |
| MP-/AB+ | 1 | 31662 | 618391 | 358230 | 666980 | 171101 |
|  | 2 | 7942 | 593945 | 298696 | 418818 | 37941 |
|  | 3 | 21108 | 578163 | 371374 | 357308 | 139729 |
| MP+/AB- | 1 | 20819 | 660858 | 356677 | 777204 | 24000 |
|  | 2 | 18059 | 582876 | 464951 | 866178 | 32000 |
|  | 3 | 21349 | 509718 | 267082 | 549600 | 20000 |
| MP+/AB- | 1 | 23891 | 495765 | 335061 | 401678 | 112000 |
|  | 2 | 28252 | 524161 | 228717 | 539912 | 324000 |
|  | 3 | 38019 | 599040 | 440924 | 471470 | 340000 |

Table S2. Sequencing depth obtained from each sample after sequence treatment and quality filtering using different sequencing approaches.

| **MAG** | **Completion** | **Redundancy** | **Size (Mbp)** | **Taxonomy estimation** |
| --- | --- | --- | --- | --- |
| *Achromobacter* | 94.4% (ILL)  64.8% (OP) | 7% (ILL)  1.4% (OP) | 5.92 (ILL)  2.58 (OP) | *Achromobacter sp. 002902905* |
| *Acinetobacter* | 78.9% (ILL) | 2% (ILL) | 2.38 (ILL) | *Acinetobacter* |
| *Aeromonas* | 78.9% (ILL)  91.6% (OP) | 7% (ILL)  2.8% (OP) | 2.45 (ILL)  3.84 (OP) | *Aeromonas* |
| *Herbaspirillum* | 88.7% (ILL)  98.6% (OP) | 5.6% (ILL)  5.6% (OP) | 4.47 (ILL)  5.6 (OP) | *Herbaspirillum* |
| *Stenotrophomonas* | 100% (ILL, OP, UC) | 4.2% (ILL)  7% (OP)  0% (UC) | 5.07 (ILL, UC)  5.47 (OP) | *Stenotrophomonas maltophilia* |
| *Comamonas* | 76.1% (OP) | 2.8% (OP) | 5.34 (OP) | *Comamonas* |
| *Chryseobacterium* | 84.5% (OP) | 4.2% (OP) | 4.35 (OP) | *Chryseobacterium* |
| *Enterobacteriaceae* | 83.1% (OP) | 0% (OP) | 4.78 (OP) | Unknown |
| *Enterobacteriaceae2* | 54.9% (OP) | 5.6% (OP) | 4.86 (OP) | Unknown |
| *Klebsiella* | 95.8% (OP) | 4.2% (OP) | 5.65 (OP) | *Klebsiella* |

Table S3. Completion, redundancy, size and taxonomy estimation of the MAGs obtained from the assembly of short-reads and the hybrid assemblies of short and long reads. ILL: short-read Illumina assembly. OP: OPERA-MS hybrid assembly. UC: Unicycler hybrid assembly.

| **MAG** | **ARGs** | **Full operon** | **Antibiotic class** | **Mechanism** | **Found in integron reads** | **Plasmid-like contig** |
| --- | --- | --- | --- | --- | --- | --- |
| *Achromobacter* | abeS | Yes | Multidrug | Antibiotic efflux | Yes | No |
|  | mexI | No | Multidrug | Antibiotic efflux | Yes | No |
|  | muxB | No | Multidrug | Antibiotic efflux | No | No |
|  | msbA | Yes | Nitroimidazol | Antibiotic efflux | No | No |
|  | OXA-114a | - | Beta-lactams | Antibiotic inactivation | No | No |
|  | pp-flo | Yes | Phenicol | Antibiotic efflux | No | No |
| *Acinetobacter* | OXA-421 | - | Beta-lactams | Antibiotic inactivation | No | No |
|  | adeIJK | Yes | Multidrug | Antibiotic efflux | No | No |
|  | ADC-19 | - | Beta-lactams | Antibiotic inactivation | No | No |
|  | OprD | - | Imipenem | Outer membrane protein | No | No |
|  | adeAB | No | Tetracycline | Antibiotic efflux | Yes | No |
|  | ANT(3")-IIa | - | Aminoglycosides | Antibiotic inactivation | No | No |
| *Aeromonas* | OXA-12 | - | Beta-lactams | Antibiotic inactivation | No | No |
|  | MOX-7 | - | Beta-lactams | Antibiotic inactivation | No | No |
|  | fosA | - | Phosphonic acid antibiotic | Antibiotic inactivation | No | No |
|  | MCR-3 | - | Peptide antibiotic | Antibiotic target alteration | No | No |
| *Herbaspirillum* | muxB | No | Multidrug | Antibiotic efflux | No | No |
|  | msbA | Yes | Nitroimidazol | Antibiotic efflux | No | No |
| *Stenotrophom!onas* | L1 | No | Beta-lactams | Antibiotic inactivation | Yes | No |
|  | emrE | Yes | Multidrug | Antibiotic efflux | No | No |
|  | mexK | No | Multidrug | Antibiotic efflux | Yes | No |
| *Comamonas* | OXA-3 | - | Beta-lactams | Antibiotic inactivation | No | No |
|  | TEM-148 | - | Beta-lactams | Antibiotic inactivation | No | No |
| *Chryseobacterium* | - | - | - | - | - | - |
| Enterobacteriaceae | msbA | Yes | Nitroimidazol | Antibiotic efflux | No | No |

Table S4. Characterization and genetic context of the ARGs not related to ciprofloxacin and gentamicin resistance found in the MAGs.

| **MAG** | **Type IV secretion system** | **Virulence** | **Plastic degradation** |
| --- | --- | --- | --- |
| *Achromobacter* | + | + | - |
| *Acinetobacter* | + | - | 1 (low-density polyethylene) |
| *Aeromonas* | + | + | - |
| *Herbaspirillum* | + | + | - |
| *Stenotrophomonas* | + | + | - |
| *Comamonas* | + | + | - |
| *Chryseobacterium* | + | + | - |
| *Enterobacteriaceae* | + | + | - |

Table S5. Characterization of MAGs obtained from the co-assembly of short reads and the hybrid assembly of short and long reads in terms of presence (+) or absence (-) of genes involved in conjugative transfer (Type IV secretion system) and virulence.

| **Condition** | **Number of plasmid contigs** | **Size range** | **ARGs in plasmids** |
| --- | --- | --- | --- |
| MP- AB- | 11 | 1.2-39 kb | 1 *(emrE)* – resistance to macrolides |
| MP- AB+ | 10 | 1.5-45 kb | 0 |
| MP+ AB- | 8 | 1.8-39 kb | 0 |
| MP+ AB+ | 11 | 1.2-59.8 kb | 0 |
| All | 18 | 1.2-39.2 kb | 0 |

Table S6. Number of plasmid contigs, size range and number of ARGs in plasmid contigs obtained from the plasmid assembly of triplicates from each condition using metaPlasmidSPAdes. Plasmid contigs were obtained from the assembly of Illumina short-reads. No ARG-containing plasmid contigs were obtained from the hybrid assembly.

| **Condition** | **ARG-containing viral contigs from the short-read assembly** | **ARG-containing viral contigs from the hybrid assembly** |
| --- | --- | --- |
| MP- AB- | 6 | 2 |
| MP- AB+ | 0 | 1 |
| MP+ AB- | 0 | 0 |
| MP+ AB+ | 0 | 0 |
| All | 3 | 1 |

Table S7. Number of ARG-containing viral contigs obtained from the assembly of short reads and the hybrid assembly of short and long reads using metaViralSPAdes.

| **MAG** | **Viral contig 1** | **Viral contig 2** | **Viral contig 3** |
| --- | --- | --- | --- |
| *Achromobacter* | 0 | 0 | 1 (99.8% ID, 24835 of 38066 nt) |
| *Acinetobacter* | 0 | 0 | 0 |
| *Aeromonas* | 0 | 0 | 0 |
| *Herbaspirillum* | 0 | 0 | 0 |
| *Stenotrophomonas* | 1 (100% ID, 232259 of 232259 nt) | 1 (99.995% ID, 115208 of 115306 nt) | 0 |
| *Comamonas* | 0 | 0 | 0 |
| *Chryseobacterium* | 0 | 0 | 0 |
| *Enterobacteriaceae* | 0 | 0 | 0 |

Table S8. Alignment between the MAGs and the viral contigs identified in this study. ID = identity. Nt = nucleotides.

| **Genus** | **Analyzed reference genomes** | **CPX resistance genes** | **GM resistance genes** | **CPX+GM resistance genes** |
| --- | --- | --- | --- | --- |
| *Acinetobacter* | 31 | 31 (100%) | 0 | 0 |
| *Herbaspirillum* | 5 | 5 (100%) | 0 | 0 |
| *Stenotrophomonas* | 5 | 5 100%) | 0 | 0 |
| *Klebsiella* | 10 | 8 (80%) | 2 (20%) | 0 |
| *Achromobacter* | 4 | 1 (25%) | 3 (75%) | 0 |
| *Chryseobacterium* | 23 | 0 | 0 | 0 |
| *Aeromonas* | 11 | 11 (100%) | 0 | 0 |
| *Comamonas* | 9 | 4 (44%) | 0 | 1 (11%) |

Table S9. Ciprofloxacin and gentamicin resistance genes found in reference genomes of the taxa associated to the key MAGs identified in this study. The number of analyzed reference genomes represent the number of complete reference genomes found in the NCBI database.

**Figure S1. OD_600_ of 150 µl of Rhône river water bacteria incubated in 1:10 TSB in a plate reader for 42 hours in the absence of antibiotics, with gentamicin or with ciprofloxacin at 100 ng/ml.** After 42 hours, the optical density of triplicates polluted with antibiotics was compared to that of non-polluted controls using ANOVA (*p-*value = 0.2) and Dunnett’s multiple comparison tests (non-significant at a *p*-value threshold of 0.05). Thus, ciprofloxacin and gentamicin at 100 ng/ml were considered to be sub-inhibitory to Rhône river water at an overall level *in vitro.*


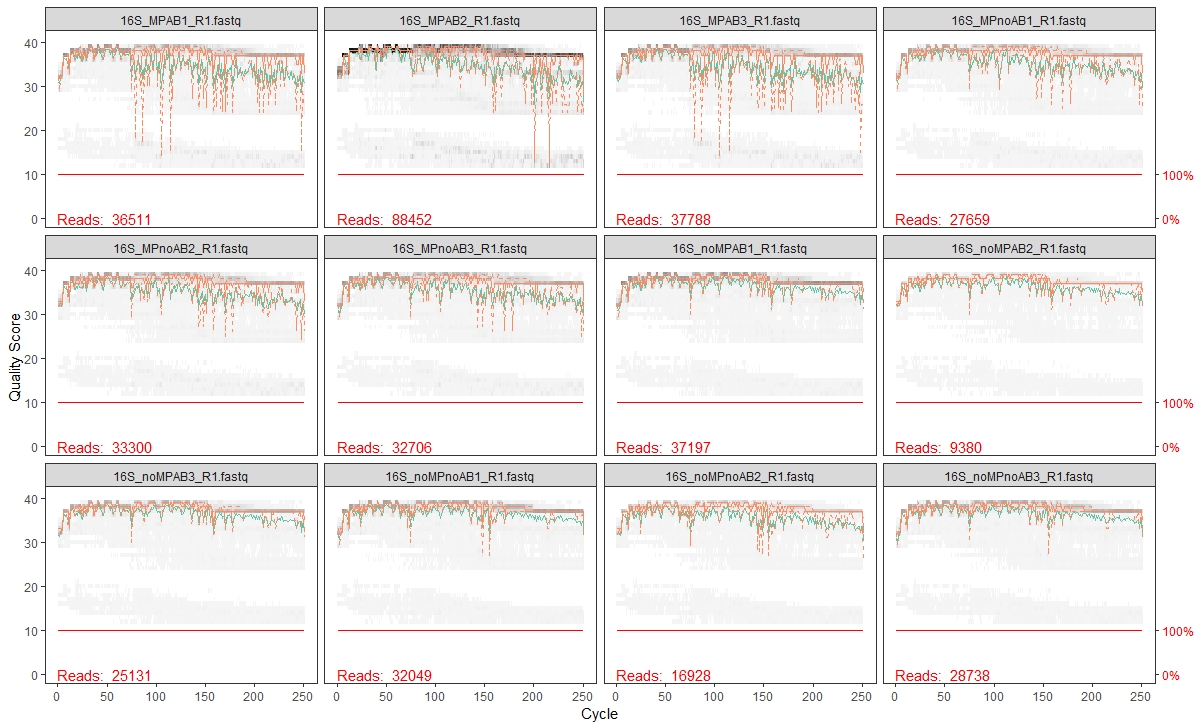


**Figure S2a. Quality score of 16S rRNA gene forward reads.**


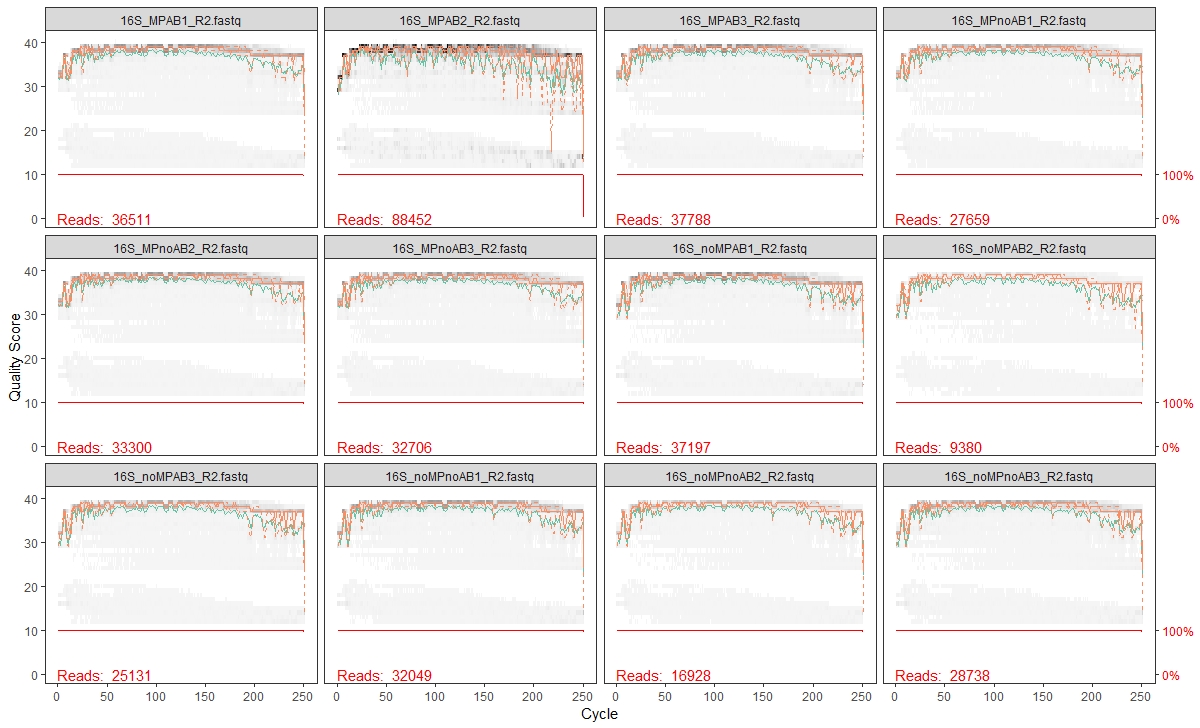


**Figure S2b. Quality score of 16S rRNA gene reverse reads.**


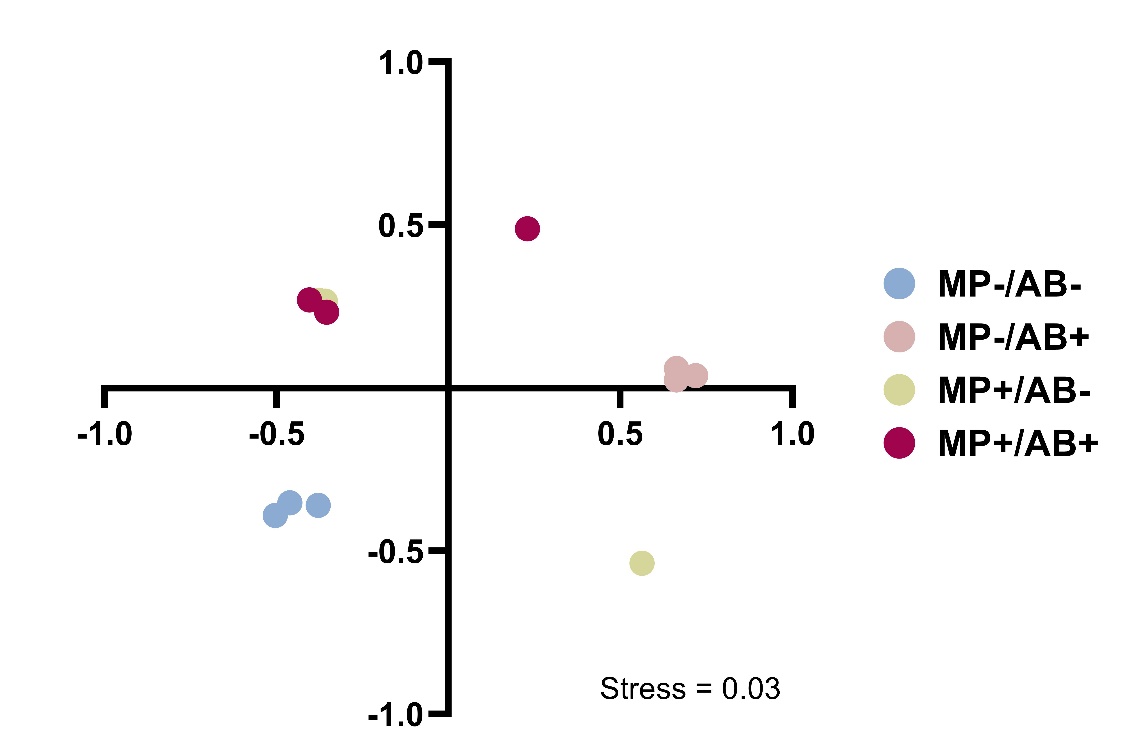


**Figure S3. NMDS of bacterial community composition of freshwater samples exposed to antibiotics, microplastics or both.** Based on the Bray-Curtis dissimilarity matrix calculated from ASVs of the 16S rRNA gene annotated to the class level. All analyses were done using the vegan package in R.

**Figure S4. Relative abundance of the MAGs obtained from the short-read co-assembly and from the hybrid co-assembly of short and long reads.** A: *Achromobacter sp. 002902905* (OPERA-MS hybrid assembly). B: *Herbaspirillum* (MEGAHIT short-read assembly). C: *Aeromonas* (MEGAHIT short-read assembly). The percent of recruitment represents the percentage of reads from a sample that map onto a MAG and is thus normalized by sequencing depth. ANOVA *p*-values: 0.0002 (A), 0.0004 (B), <0.0001 (C). Only pairwise comparisons with *p*-value < 0.05 are shown. n=3.

**Figure S5. Relative abundance of the *Stenotrophomonas maltophilia* MAGs obtained from the short-read co-assembly and from the hybrid co-assembly of short and long reads.** A: MEGAHIT short-read assembly. B: OPERA-MS hybrid assembly. The percent of recruitment represents the percentage of reads from a sample that map onto a MAG and is thus normalized by sequencing depth. ANOVA *p*-values: <0.0001 (A,B). Only pairwise comparisons with *p*-value < 0.05 are shown. n=3.

**Figure S6. Relative abundance of the two MAGs obtained from the hybrid co-assembly of short and long reads using OPERA-MS.** A: unknown genus from the *Enterobacteriaceae* family. B: *Klebsiella*. The percent of recruitment represents the percentage of reads from a sample that map onto a MAG and is thus normalized by sequencing depth. ANOVA *p*-values: <0.0001 (A,B). Only pairwise comparisons with *p*-value < 0.05 are shown. n=3.
